# Advances in Grape Seed Oil Extraction Techniques and Their Applications in Food Products: A Comprehensive Review and Bibliometric Analysis

**DOI:** 10.3390/foods13223561

**Published:** 2024-11-07

**Authors:** Jaime Laqui-Estaña, Elías Obreque-Slier, Nidia García-Nauto, Erick Saldaña

**Affiliations:** 1 Faculdade de Engenharia de Alimentos, Universidade de Campinas, rua Monteiro Lobato, 80, Campinas 13083-862, Brazil; j272329@dac.unicamp.br (J.L.-E.); ngarcian@unam.edu.pe (N.G.-N.); 2 Facultad de Ciencias Agropecuarias, Universidad Nacional de Trujillo, Av. Juan Pablo II s/n, Trujillo 13011, Peru; 3 Department of Agro–Industry and Enology, Faculty of Agronomical Sciences, University of Chile, Santiago P.O. Box 1004, Chile; eobreque@uchile.cl; 4 Escuela Profesional de Ingeniería Agroindustrial, Universidad Nacional de Moquegua, Calle Ancash s/n, Moquegua 18001, Peru; 5 Sensory Analysis and Consumer Study Group, Escuela Profesional de Ingeniería Agroindustrial, Universidad Nacional de Moquegua, Prolongación Calle Ancash s/n, Moquegua 18001, Peru

**Keywords:** *Vitis vinifera*, byproducts, vegetable oil, traditional and alternative extraction methods, food additive

## Abstract

Global wine production has grown, resulting in an increase in waste within the industry. This has raised concerns among producers and scientists worldwide, prompting them to seek solutions for its management. The aim is to explore the latest advancements in using grape seed oil as a byproduct and its applications within the food industry. To achieve this, a bibliometric analysis was conducted using the Scopus database covering the period from 1990 to 2023. Additionally, a comprehensive literature review was conducted on extraction techniques, compositions, properties, and innovative applications in food. A bibliometric analysis revealed that interest in grape seed oil has grown over the past fifteen years. The majority of research on this grape byproduct is concentrated in Asian countries. Grape seed oil is a rich source of lipophilic compounds, including fatty acids, phytosterols, and vitamin E, which provide antioxidant and antimicrobial properties. The literature indicates that only oil obtained through pressing is used in food products, such as meat products, dairy drinks, and chocolates, either directly or in emulsions. These findings suggest that further research and innovation are needed to explore how this waste can be used in new food sources, particularly in countries with high wine production.

## 1. Introduction

**Figure 1:**
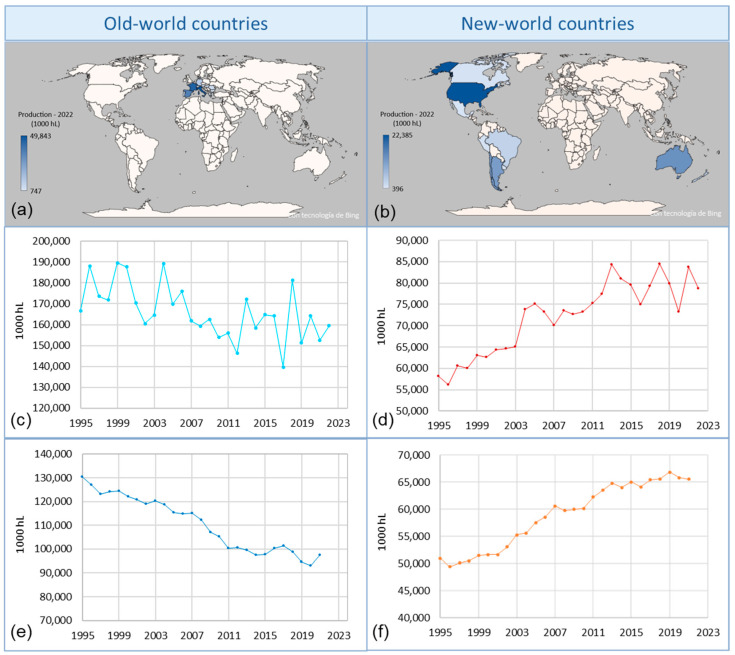
Wine production by country (**a**,**b**), global wine production (**c**,**d**), and global wine consumption (**e**,**f**) according to the OIV in countries of the old-world and new-world (data extracted from the OIV on 25 August 2023).

According to the International Organization of Vine and Wine [1], wine production has slightly increased globally in recent years. This is mainly due to the wine-producing countries of the new-world (Argentina, Australia, Brazil, Canada, Chile, United States, Mexico, New Zealand, Peru, South Africa, and Uruguay) having raised their production from 58 to 78 million hL between 1995 and 2022, while the old-world wine producers (Austria, Bulgaria, France, Germany, Greece, Hungary, Italy, Portugal, Romania, Spain, and Switzerland) have experienced a slight decrease from 166 to 159 million hL [2]. Therefore, as shown in Figure 1, the rise in wine consumption in the new-world countries has contributed to this slight increase in global wine production [1].

Winemaking generates waste like grape pomace, which consists of skins, seeds, and stems. This waste is utilized as organic fertilizer [3], but improper management can lead to environmental pollution and financial losses for wineries [4]. Grape seeds are a byproduct of the wine industry, and due to their bioactive compounds, they can be considered as a valuable resource for recovery [5,6].

In *Vitis vinifera* species, each grape berry contains four seeds, representing 3–6% of the fresh weight of the berry, or 38–52% of the dry residue of winemaking [7,8], containing water (25–45%), carbohydrates (35%), oil (13–20%), tannins (4–6%), nitrogenous compounds (4–6%), and mineral materials (2–4%) [9,10]. Researchers have shown considerable interest in grape seed oil due to its high concentration of bioactive compounds that can be used as a source of natural antioxidants and antimicrobials [11,12] in food and non-food sources [13,14].

Grape seed oil has both odorless and aroma compounds related to wine [15,16]. These characteristics make it an attractive option for various applications in the food industry. Therefore, this review aims to conduct a bibliometric analysis and narrative review on the current interest of researchers in grape seed oil and its composition, properties, and applications in the food industry based on the extraction methods used.

## 2. Materials and Methods

Two methodologies were employed to extract and process the data. The first methodology involved a bibliometric analysis, while the second methodology was a narrative review. For the bibliometric analysis, we searched for relevant documents in the Scopus database on 24 August 2023 using the keywords grape AND seed AND oil. This search yielded 1268 documents, which were narrowed down to 611 documents by refining the search with quotes around the words grape seed oil. Further refinement was achieved by limiting the search to documents that included those words in the article title, which narrowed it down to 235 documents. Finally, we only considered original articles published between 1990 and 2023, resulting in 198 articles for analysis. We downloaded these articles in a Microsoft Excel format and processed them using VOSviewer 1.6.18 software [17] to build and display graphical representations of bibliometric maps. To obtain these co-occurrence keywords plots, we cleaned the data by eliminating irrelevant keywords such as article and priority journal, lemmatizing keywords with the same root, and replacing the first keyword with the second. A threshold of 7 was established as the minimum number of occurrences of a keyword, resulting in 49 keywords meeting the criterion.

Likewise, the 198 documents were also analyzed using the R software and the R-package Bibliometrix, version 4.2.3 [18], to visualize the annual production of scientific documents and word clouds of frequencies for the last three decades. Lastly, we analyzed the Scopus database to determine the production of documents by country. This methodology served as a guide to carry out the narrative review.

For the narrative review, we only considered documents from the Scopus database published between 2006 and August 2023. The keywords used for the search were “grape seed oil” OR “winemaking byproducts” AND “grapeseed oil uses” AND “oil extraction techniques” AND “grapeseed oil properties”, searching for these keywords in the article title, abstract, and keywords section. The search included all types of publications, such as original articles, reviews, book chapters, and web pages, and it excluded studies unrelated to the keywords.

## 3. Results

### 3.1. Bibliometric Approach

**Figure 2:**
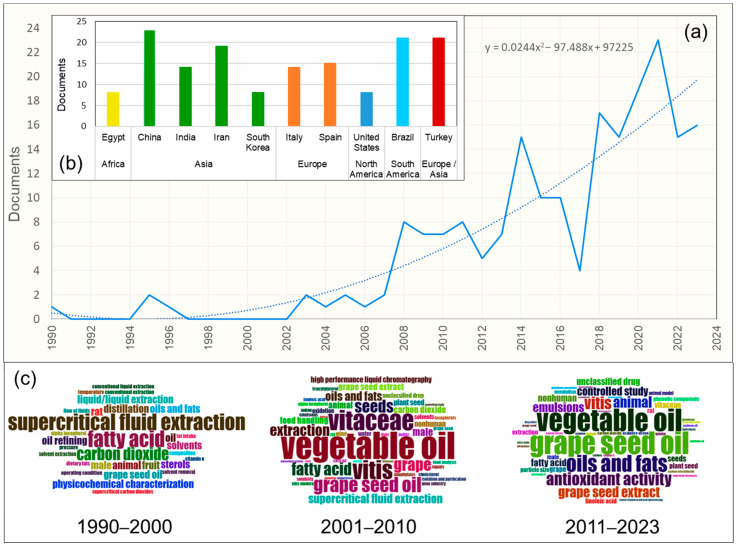
Production of scientific documents obtained from the Bibliometrix website (**a**) and by country. Figure obtained from Scopus (**b**). The lower part the word cloud by period was sourced from the Bibliometrix website (**c**) (data extracted from Scopus on 24 August 2023).

Figure 2 illustrates the growth in scientific publications starting from 2008 according to the bibliometric analysis. The analysis also reveals that Asian countries are more interested in researching grape seed oil, despite not being wine-producing countries, compared to wine-producing countries [1]. This finding should encourage academic institutions and wine-producing countries to increase research funding for the comprehensive utilization of this waste. The word cloud by period displays the frequencies of the keyword ‘plus’ from 1990 to 2023. The initial focus of interest was studying non-conventional oil extraction methods using supercritical fluids containing carbon dioxide (CO_2_). Later, the scientific focus shifted to studying vegetable oils, particularly of the genus *Vitis*. Finally, it can be seen that the most critical topic of interest in the last decade has been studying grape seed oil and its properties.

**Figure 3:**
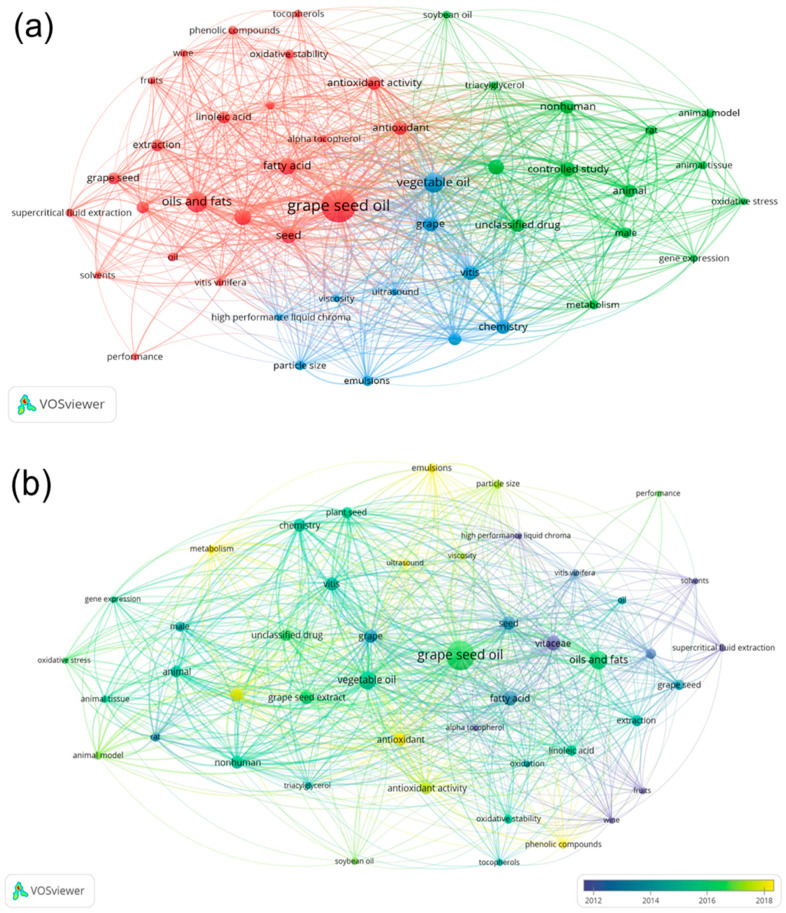
Clusterization map (**a**). Co-occurrences of the keywords over time (**b**). Figures obtained using VOSviewer software (data extracted from Scopus on 24 August 2023).

The next figure presents maps of clustering and co-occurrences showing three clusters of keywords. In Figure 3a, the red cluster highlights the relationships between grape seed oil and the extraction process, composition, and properties. The green cluster depicts the relationships between grape seed extracts and case-controlled and animal studies, and finally, the blue cluster shows the relationships between vegetable oil physical and chemical characteristics. In Figure 3b, the current research topics related to grape seed oil such as phenolic compounds, antioxidant activity, emulsions, ultrasound, metabolism, and controlled studies are illustrated. These topics indicate the current trends of studying the composition, properties, animal studies, and applicability of grape seed oil in various forms such as oil or the formation of emulsions.

Based on the bibliometric analysis, it can be inferred that the study of grape seed oil, its composition, properties, and potential uses in various industries has gained significant interest in academia since 2008. These data served as a starting point for conducting an extensive narrative literature analysis, focusing on the most relevant research topics related to this byproduct of winemaking.

### 3.2. Narrative Review

#### 3.2.1. Byproduct of Winemaking

A significant amount of solid waste is generated when making wine, known as pomace. Although grape pomace is a byproduct of low economic value, it contains a considerable number of compounds with high added value. These pomaces are composed of grape stems (24.9–25.0%), seeds (22.5–25.0%), and skins (42.5–50%) [19,20]. Since grape seeds are discarded as part of the winemaking process, extracting and selling grape seed oil and extract could be a profitable secondary activity that could efficiently use this byproduct.

#### 3.2.2. Process of Drying and Grinding of Grape Seeds

**Figure 4:**
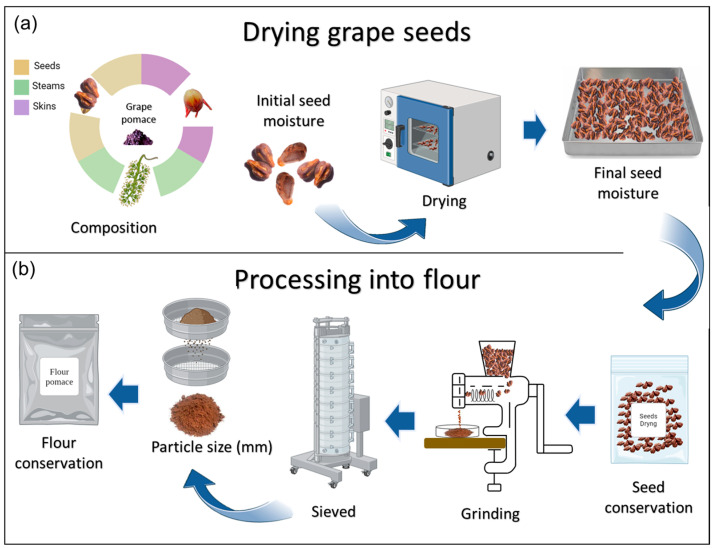
Grape seed drying process (**a**) and grinding processing into flour (**b**).

Prior to the extraction process of grape seed oil, grape seeds must undergo a series of drying (Figure 4a) and grinding processes (Figure 4b). Initially, the seeds are separated from the skins manually or mechanically. Then, the seeds are dried in a drying chamber (oven) at 55 °C for 48 h [21,22], until reaching a constant weight with less than 10% moisture. A study found that in oils extracted using the Soxhlet and Soxtherm apparatus, reducing the moisture content of the seeds from 10 to 2.5% increased the yield by nearly 1% [23]. A second procedure was carried out by drying the pomace (seeds and skins) in a drying chamber until a constant weight of <10% moisture was obtained; subsequently, the researchers separated the seeds from the skins using vibrating sieves [21,22,24]. After dehydrating the seeds using one of these two procedures, they were placed in vacuum-sealed bags and stored in darkness at room temperature [15,22,25], or −18 or −20 °C [26,27]. The dried seeds were then ground into a powder with a particle size of less than 0.5 mm (indicated by several researchers later on). Reducing particle size increases the surface area per unit volume, enhancing oil diffusion. In one study, it was observed that decreasing the particle size from 0.75 to 0.41 mm increased the yield from 8 to 16% [22]. This sieving process is part of the characterization but will not be included in the final procedure. This stage allows for the separation of particles of different sizes using sieves or meshes with specific openings, resulting in finer particles or particles of uniform size. This could potentially affect the oil extraction process. However, further studies are recommended to specifically examine the impact of particle size on the obtained grape seed oil. Finally, the grape seed flour is stored in vacuum-sealed polypropylene bags at −20 °C without light [27,28]. Considering the drying and grinding stages before the extraction process could enhance the yield. Therefore, optimization studies for the parameters at each stage are recommended.

The following details the stages of the drying and grinding process, including values reported by various authors. Initial seed moisture (%): 43–49 [25] and 25–45 [19]. Drying of seeds: 50 °C for 24 h [15], 40 °C for 20 h [26], 54 °C for 48 h [27,29], 24 °C for 96 h [30], and 35 °C for 48 h [31]. Final seed moisture (%): 1.5–2.0 [27], 2.5 [23], 5–10 [25], 7.0 [32], 7–8 [33], 8.0 [31], 8.3 [34], 9.38 [35], and <10 [36]. Seed conservation: room temperature [15,22,25] or –18 to −20 °C [26,27]. Seed grinding: steel grinder [34], screw grinder [37], coffee grinder [36,38], vibratory disc mill [15], Moulinex A320 R grinder [23], electric grinder HR2185 Philips [39], or grinder with integrated cooling system [31]. Particle size after sieving (mm): 0.25–0.43 [15], 0.38 [35], 0.41 [22], 0.5 [23,26,27,29,34], and 0.61 [37]. Flour conservation: vacuum sealed in polypropylene bags at −20 °C [27,28].

#### 3.2.3. Grape Seed Oil Extraction Process

**Figure 5:**
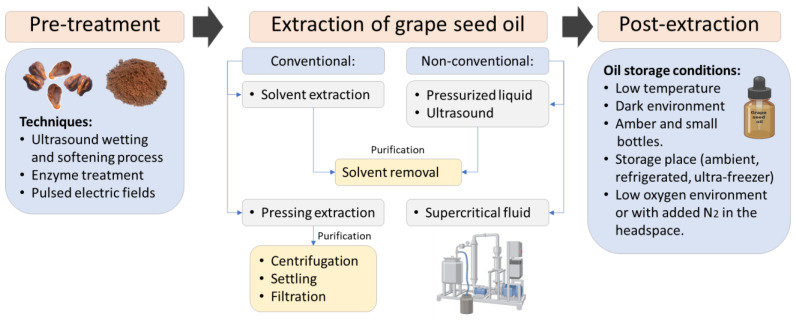
Grape seed oil extraction process stages.

Pre-treatment: Since conventional grape seed oil extraction methods present certain limitations regarding extraction yield and solvent use, it is necessary to resort to pre-treatment techniques to improve the extraction yield and the quality of the oil obtained. Below are some reports of the pretreatments used (Figure 5); for example, ultrasonic pretreatment before the Bligh Dyer and supercritical fluid CO_2_ extraction, where the seeds were immersed in a polyethylene package in an ultrasonic bath at 30 °C for 30 min of sonication (best condition for pre-treatment), which contributed to increasing the content of *α*-tocopherol about 56 and 99% in Bligh Dyer and supercritical fluids, respectively [26]. In another study, it was observed that before extraction via pressing, by using various optimized parameters (amount of enzyme additive, hydrolysis temperature, hydrolysis time, degree of crushing, screw speed, squeezed water), with 1 g of added enzyme (protease and cellulase), 60 mesh, and 8% squeezed water, it was possible to reduce the percentage of waste in the process of obtaining oil via pressing from 33.24 to 57.79% [40]. A recent study employed a pretreatment using pulsed electric fields (PEFs) before extraction with supercritical fluids. This pretreatment with PEFs increased the extraction yield (with values ranging from 78.4 to 81.8 g/kg) compared to extraction using supercritical fluids (ranging from 76.3 to 78.6 g/kg) and cold pressing (67.1 g/kg). Furthermore, the extraction of sterols and non-flavonoid phenolic compounds saw significant enhancements, reaching 5347.0 and 1378.3 mg/kg, respectively, under optimized conditions for supercritical fluid extraction with CO_2_ (35 MPa and 45 °C) and pulsed electric fields (5 kV/cm at 120 Hz for 5 min) [31].

Extraction. Grape seed oil can be obtained either through direct pressing of whole seeds or from grape seed flour. Conventional methods for oil extraction involve mechanical pressing and solvent extraction techniques. Furthermore, alternative methods such as ultrasound-assisted extraction [34,36,40], supercritical fluids with CO_2_ [26,27,31,35], and supercritical fluids with CO_2_ + ethanol [27] are available (Figure 5). Below are explanations of the various methods employed for extracting grape seed oil.

Pressing is one of the oldest oil extraction techniques. Research shows that various presses have been used to extract oil directly from whole grape seeds, including screw presses [32,40,41,42,43,44] and hydraulic piston presses [36,45]. Additionally, screw presses [46] and hydraulic presses [47] have been utilized to extract oil from grape seed flour. However, the screw pressing method can increase the oil temperature by 40 to 50 °C as a result of the pressure and friction generated by the screws [32]. This temperature increase can affect the oil’s antimicrobial and antioxidant properties. Despite its low yield, this method has the advantage of avoiding solvent use during extraction, making it a reliable and desirable option for consumers in food products [48,49,50].

Solvent extraction is the most widely used method for extracting oil from seeds. Its main advantage is its speed, cost-effectiveness, and high yield due to the long contact time between the seeds and the solvent. However, since it involves organic solvents, the final purification process can be expensive and hazardous due to the toxicity of the solvents involved, such as ethanol, methanol, hexane, or petroleum ether. There is a growing trend towards using ethanol as a solvent, as it is safe for consumption once the extraction chemicals have been removed, and it is considered food-grade [51].

Ultrasound-assisted extraction is a method that leverages the cavitation phenomenon, which is the production and rupture of microscopic bubbles in the food matrix. This process causes the cell membrane to degrade, leading to a rapid extraction rate of the extractable compounds [51]. This method has been widely used with organic solvents to extract compounds quickly and efficiently [34,36,52,53].

The pressured liquid extraction method is a quick and efficient technique for extracting analytes from different types of samples using liquid solvents at different temperatures and pressures [54]. A key advantage of pressurized liquid extraction over pressing and solvent extraction is that solvents under pressure remain liquid, even at temperatures above their normal boiling points, facilitating high-temperature extraction. Therefore, the application of this method resulted in an enhancement of the vitamin E content in grape seed oil [47].

Supercritical fluid extraction involves placing solid materials in a container and gradually adding supercritical fluids until the required extraction conditions are reached. CO_2_ is commonly used for this process due to its non-toxicity, low cost, absence of odor and taste, and ease of removal without leaving any residues [55]. This fluid exhibits excellent solvent properties for nonpolar and some polar molecules [51]. To achieve higher yields and antioxidant activity, the extraction using supercritical CO_2_ has been optimized at 400 bar pressure and 41 °C [35]. Recently, CO_2_-expanded ethanol has been used to increase the extraction yield and isolate compounds from grape seed oil. The optimal conditions for this method are a pressure of 7.4 MPa, a temperature of 40.85 °C, and a CO_2_ mole fraction of 0.3 [27].

Post-extraction: After the grape seed oil is extracted, it undergoes purification using various techniques, and it is stored for analysis or application. Different papers describe the storage conditions used for different extraction methods. For instance, if the oil is obtained through pressing, it must undergo purification techniques such as centrifugation, filtration, or natural decantation before storage. The purified oil is stored in small, amber-colored vials at 2 to 6 °C [32,56,57,58]. Different authors have proposed storage conditions such as storing the oil at −18 and −20 °C with the addition of gaseous nitrogen (N_2_) directly into the headspace [16,31] or without the addition of N_2_ [42]. Oils obtained with solvents are kept in amber-colored bottles stored at 10 °C [15] or under inert atmospheric conditions with the addition of N_2_ at temperatures of −18 to −20 °C [26,27,59] or −55 °C [33]. Oils obtained using supercritical fluids with CO_2_ are stored at −18 and −20 °C in a dark place [27,31] or in amber vials at room temperature before analysis [29]. After analyzing the published scientific evidence, it is clear that the extraction technique used can significantly affect the storage conditions of the oil. The less harsh the extraction method, the more antioxidants and beneficial compounds the oil tends to retain. Consequently, these oils are usually more sensitive to light, heat, and oxygen and require careful storage to preserve their quality.

#### 3.2.4. Yield and Composition of Grape Seed Oil

**Figure 6:**
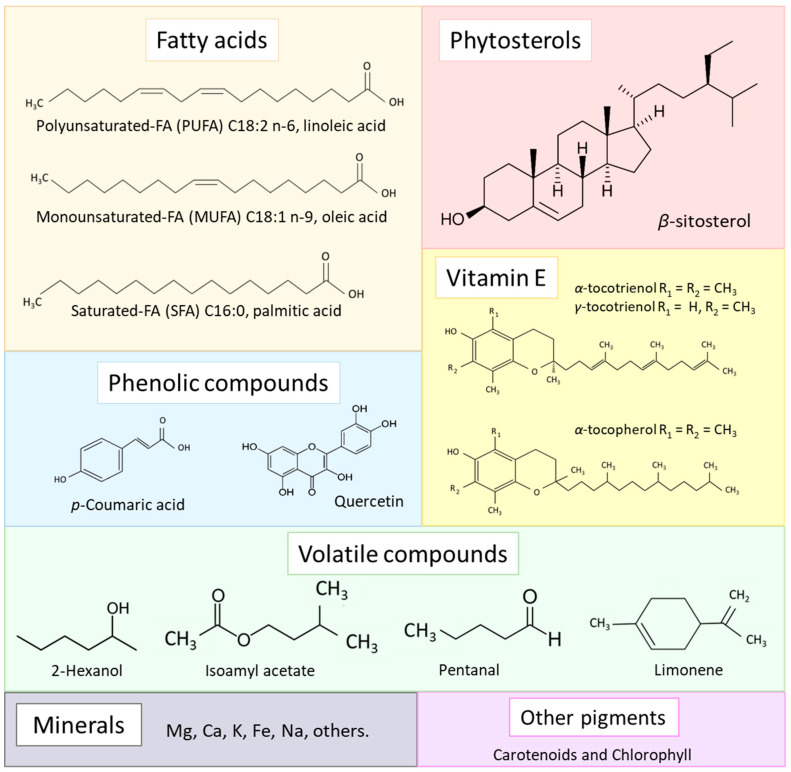
Main compounds of grape seed oil.

The extraction yield of grape seed oil varies from 3.9 to 18.5% (g of oil per 100 g of dry weight of byproduct) depending on the method used. The pressing method provides the lowest yields, while the Soxhlet method and the method using supercritical fluids provide the highest yields (see Supplementary Materials). The yield depends on extrinsic factors such as the extraction technique, type of solvent, operating conditions, and environmental aspects of the grape crop, as well as intrinsic factors such as grape variety. Grape seed oil is composed mainly of triglycerides (usually about 99%) and unsaponifiable materials (typically 1%). Triglycerides are ester derivatives of glycerol and fatty acids. The main unsaponifiable compounds include phytosterols, phenols, vitamin E, and others (refer to Figure 6) [13,60].

**Figure 7:**
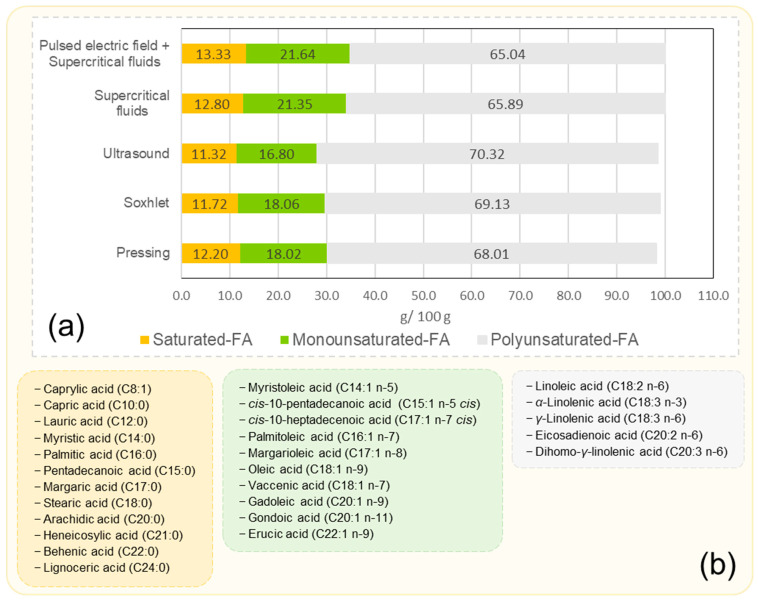
Profile of fatty acids in grape seed oil according to the extraction method (**a**), classification of the fatty acids according to the degree of unsaturation; saturated fatty acids (yellow color), monounsaturated fatty acids (green color), and polyunsaturated fatty acids (gray color) (**b**).

Fatty acids: It is important to note that grape seed oil is mainly made up of polyunsaturated fatty acids (PUFA), followed by monounsaturated fatty acids (MUFA) and saturated fatty acids (SFA), regardless of the extraction method used. The data were obtained from the averages of studies using the pressing method [30,31,36,41,44,52], Soxhlet [28,30,39,59,61,62,63], ultrasound [36,52,53], supercritical fluids [31,64], and pulsed electric field combined with supercritical fluids [31] (Figure 7a). Understanding this is crucial because the oxidation of fatty acids is correlated with the content of polyunsaturated fatty acids; oils with high PUFA contents are more prone to oxidation [65]. Figure 7b shows the classification of fatty acids found in grape seed oil based on the degree of unsaturation [26,30,33,59,66,67,68,69,70].

In the various studies reviewed, regardless of the type or extraction method used, linoleic acid comprised most of the oil, ranging from 57.68 to 74.82%, followed by oleic acid at 6.34 to 24.9% and palmitic acid at 6.26 to 23.5%. Meanwhile, stearic acid ranged from 2.80 to 11.04%, *α*-linolenic acid ranged from 0.0 to 0.64%, and palmitoleic acid ranged from 0.0 to 0.37%, as shown in Table 1. Furthermore, this table demonstrates the influence of the extraction method on the fatty acid profile, with no clear differences. However, oils obtained via pressing exhibit slightly higher oleic acid values, while those obtained via Soxhlet show slightly higher palmitic acid values. Regarding the influence of the variety, it was observed that the Pinot noir and Merlot varieties had slightly higher concentrations of linoleic acid and lower concentrations of α-linolenic acid. These results suggest that optimizing the extraction methods based on the variety could lead to more desirable specific fatty acid profiles.

On the other hand, the amount of linoleic acid in grape seed oil was similar to that observed in other species, e.g., safflower 79.1%, heglig (*Balanites aegyptiaca*) 75.86%, thistle (*Silybum marianum*) 63.3%, hemp 50–70%, sunflower 62.2%, evening primrose (*Oenothera* spp.) 65–80%, walnut 59.7%, corn 53.5%, wheat germ 59.7%, and pumpkin seed oil 49–69% [71,72,73]. This is important because studies have shown that a moderate intake of linoleic acid (18:2 n-6), about 4.4 to 6.7 g per day based on a 2000-calorie diet for adults, along with a decrease in total and saturated fat intake, may beneficially influence lipoprotein metabolism, lower blood pressure, and reduce the risk of cardiovascular disease [73,74,75]. Similarly, the *α*-linolenic acid content in grape seed oil was observed to be similar to other species, e.g., safflower 0.15%, thistle (*Silybum marianum*) 0.88%, hemp 0.36%, sunflower 0.16–0.5%, sesame 0.21%, rapeseed 0.45%, olive 0.6%, and pumpkin seed oil 0.12% [71,76]. However, it was lower compared to other species, e.g., linseed, 55.3–59.39%, and canola, 8.6–12.21% [77,78].

Additionally, it is important to note that linoleic (n-6) and *α*-linolenic acid (n-3) are considered “essential” fatty acids; the human body does not synthesize these, and they must be obtained primarily through the diet. However, it is essential to note that the n-3/n-6 ratio in grape seed oil is relatively high, ranging from 147 to 235 in a study of eighteen grape varieties [39]. These values are similar to those found in other species, such as sunflower seed (131) and sesame (113) [76], but they are significantly higher compared to species like hemp seed oil (3.29), flax (0.28), canola (1.97), olive (16), soybean (6.7), and mustard (2.2) [76,78]. Studies suggest that a high n-3/n-6 ratio may be harmful to human health, whereas a ratio closer to 1:1 is associated with a lower risk of chronic diseases [79].

Phytosterols: Phytosterols are lipophilic compounds of plant origin. They are recognized for their ability to reduce cholesterol, and they are added to foods in free or esterified form [80]. Grape seed oil is a valuable source of various phytosterols. It is relevant to note that 16 phytosterol compounds have been found in grape seed oil (brassicasterol, campesterol, campestanol, stigmasterol, Δ7-campesterol, clerosterol, *β*-sitosterol, sitostanol, Δ5-avenasterol, Δ5,23-stigmastadienol, Δ5,24-stigmastadienol, Δ7-stigmasterol, Δ7-avenasterol, cholesterol, 24-methylene cholesterol, and squalene), regardless of the variety. The phytosterols with the highest reported proportions in grape seed oil were *β*-sitosterol, followed by stigmasterol and campesterol, in that order, regardless of the extraction method used-whether pressing [31,44,46,81], Soxhlet [28,82,83], or supercritical fluids [31,84,85,86]. On the other hand, employing new extraction methods such as supercritical fluids has been found to enhance the total phytosterol content in grape seed oil compared to traditional extraction techniques, e.g., the phytosterol content increased from 2417.0 to 3874.7 mg/kg of oil (pressing), and from 1609.9 to 3814.6 mg/kg of oil (Soxhlet), and from 4680.4 to 4926.7 mg/kg of oil using supercritical fluids [28,31].

Vitamin E: The main compounds of vitamin E found in grape seed oil are the isomers of tocopherols (*α*, *β*, *γ*, *δ*) and tocotrienols (*α*, *β*, *γ*, *δ*) (refer to Figure 6). Both are complexes of fat-soluble vitamins, consisting of a chromanol group in the central part and a prenyl side chain [87]. The tocols (tocopherols and tocotrienols) are considered a source of natural antioxidants due to their ability to counteract radicals causing lipid peroxidation [88]. In general, tocopherols are found in the green parts of the plants and tocotrienols are found in the seeds [89].

According to Table 2, it is observed that the isomers (*α* and *γ*) are the most abundant tocopherols and tocotrienols. Likewise, it can be seen that regardless of the extraction method, the grape seed oil exhibited a higher concentration of tocotrienol isomers compared to tocopherols. Alternatively, it can be noted that tocopherol and tocotrienol extraction can be enhanced through unconventional methods. For instance, extraction using supercritical fluids resulted in greater extraction of tocols (567–263 mg/kg) compared to Soxhlet extraction (248–438 mg/kg) [29]. Similarly, the amount of *α*-tocopherols obtained via ultrasound extraction (18.3–27.7 mg/kg) was higher compared to the pressing method (5.4–16.7 mg/kg) [36]. Likewise, it was observed that the optimized pretreatment using a pulsed electric field before the extraction with supercritical fluid improved the extraction of *α* and *γ* tocopherols and tocotrienols compared to extraction using supercritical liquid fluids [31]. Therefore, we can conclude that incorporating eco-friendly methods like ultrasound extraction, supercritical fluids, and pulsed electric fields as a preliminary step represents a highly favorable option for extracting vitamin E-enriched oil.

Phenolic compounds: The phenolic compounds present low solubility in oily phases; however, small amounts can be transferred from the solid parts of the seed to the oil during the extraction process [90]. Regarding the total phenol content (Table 3), grape seed oil exhibited significant variation, ranging from 0.93 to 154.0 mg GAE/100 g oil (expressed as gallic acid equivalent/g). This variability may be attributed to various factors. For example, pretreating the seeds by drying them in an oven at 50 °C for 6 h increases the phenolic content compared to drying at 20 °C for 7 days in oils obtained via pressing [81]. In the same way, refining processes (centrifugation, filtration, or decantation) of the oil obtained via pressing could partially or completely reduce the content of polyphenols, since it was observed in a study that the oil after being clarified only had small amounts of catechin, epicatechin (1.3 mg/kg), and *trans*-resveratrol (0.3 mg/kg) [91]. Likewise, the type of solvents also influences the determination of phenols in the oil, since in some investigations, a methanol/water mixture was used [29,36,44,57,58,66,92]; in others, only methanol was used [93,94]; and in others, only water was used [33,53]. According to these results, it could be indicated that this last factor (the type of solvent used) would be an important factor to consider in order to know the real content of total phenols in grape seed oil.

Additionally, Table 3 demonstrates that the traditional Soxhlet method efficiently extracts phenolic compounds, reaching up to 113.0 mg GAE/100 g oil. Conversely, the ultrasound method appears to be a promising alternative, with the ability to extract up to 154.0 mg GAE/100 g oil from white variety seeds. Notably, the ultrasound method achieved the highest levels of extracted phenolic compounds. It has also been noted that using ultrasonic pretreatment at optimized time and temperature settings (30:30), combined with supercritical CO_2_ extraction, can enhance the efficiency of extracting phenolic compounds from grape seed oil [33]. Exceptionally, one study found that the total polyphenol content in purified grape seed oil (Carignan variety), obtained via pressing, was 0.35 mg GAE/g oil, higher than the 0.15 mg GAE/g oil found in olive seed oil [38]. In other studies, the total phenol content in grape seed oil extracted via pressing was found to range between 77 mg GAE/100 g oil (var. Albariño) [90] and 123 mg GAE/100 g oil of dried sample; higher than that of almond oil (5 mg GAE/100 g oil) and pomegranate oil (54 mg GAE/100 g oil) but lower than walnut seed oil (802 mg GAE/100 g oil) [49].

Although during the extraction, numerous bioactive compounds are transferred from the grape seed to the oil, the pressing residues preserve notable concentrations of phenolic compounds that would be a valuable resource to be investigated [66,81,91,93]. That quantity may vary depending on the variety, pretreatment of the seeds to obtain the oil, oil extraction method, refining, and mainly the technique for determining the phenolic compounds. These results suggest that several factors influence the extraction of total phenolic compounds in grape seed oil.

Table 4 shows the nonflavonoid and flavonoid phenolic compounds found in grape seed oil according to the different extraction methods. Regarding flavonoids, it can be observed that the pressing method extracts a higher proportion of ursolic acid (82.3 to 136.3 µg/g) and resveratrol (2.16 µg/g). The Soxhlet method extracts *trans*-cinnamic acid (8.48 µg/g), caffeic acid (5.20 µg/g), and gallic acid (3.70 µg/g). In comparison, the supercritical fluid method extracts *trans*-cinnamic acid (20.65 µg/g), caffeic acid (5.02 µg/g), *trans*-ferulic acid (4.34 µg/g), and *p*-coumaric acid (3.14 µg/g). A comparison of the four methods shows that the pressing method yields the highest amount of resveratrol, the Soxhlet method is best for extracting gallic acid, and the supercritical fluid method is most effective for trans-ferulic acid and *p*-coumaric acid. Regarding the flavonoid phenolic compounds, the main groups with the most compounds were flavan-3-ols and flavonols. Additionally, it was observed that the pressing method extracted the greatest quantities of kaempferol (1.8 µg/g) and procyanidin dimer B1 (1.515 µg/g). The Soxhlet method extracted (−)-epicatechin (18.8 µg/g), (+)-catechin (12.22 µg/g), and formononetin (14.25 µg/g), while the supercritical fluid method extracted formononetin (108.81 µg/g), naringenin (13.77 µg/g), and quercetin-3-*β*-d-glucoside (5.17 µg/g). Comparing the four methods, the Soxhlet method achieved the highest extraction of (+)-catechin and (−)-epicatechin, whereas the supercritical fluid method was most effective for extracting quercetin-3-*β*-d-glucoside. Alternative extraction methods show potential for enhancing the recovery of phenolic compounds and increasing antioxidant content in grape seed oils. Additionally, the choice of grape variety may be key in determining the quantity and type of phenolic compounds extracted.

Other pigments: Furthermore, grape seed oil contains other pigments in smaller quantities, such as carotenoids and chlorophyll. The concentration of carotenoids varied depending on the extraction method used, from 26.7 mg/kg (Syrah) and 26.5 mg/kg (Tintorera) in virgin grape seed oil obtained through pressing [41], 338.5 to 598.5 mg/kg in commercial pressed oil [16], 2.6 mg/kg (Chardonnay) to 4.0 mg/kg (Barbera) in Soxhlet-extracted oil, and 2.7 mg/kg (Chardonnay) to 4.8 mg/kg (Barbera) in oil extracted using supercritical fluids [29]. The presence of carotenoids in grape seed oil holds significance, as they contribute to its coloration [16]. Moreover, carotenoids play a crucial role in human nutrition, as they serve as precursors to vitamin A and exhibit antioxidant properties [95].

Variations in chlorophyll pigment content were also identified based on the method of oil extraction, with levels ranging from 9.11 mg/kg (Syrah) to 9.08 mg/kg (Tintorera) in virgin grape seed oil obtained through pressing [41], 3.0–4.0 mg/kg in commercial oil obtained by pressing [16], 1.1 mg/kg (Chardonnay) to 3.2 mg/kg (Barbera) in oil extracted using the Soxhlet method, and 1.0 mg/kg (Chardonnay) to 3.8 mg/kg (Barbera) in oil extracted using supercritical fluids [29]. However, the presence of these elements in the oil reduces its quality, as they act as pro-oxidants, thereby decreasing its oxidative stability and shelf life [52]. Hence, future research faces the challenge of developing refining techniques to eliminate these compounds without removing the beneficial antioxidant compounds. Although lower values of carotenoids and chlorophyll were obtained using the Soxhlet method and supercritical fluids, it is recommended to conduct extraction studies using alternative methods and selecting specific varieties, as this could influence the carotenoid and chlorophyll contents.

Minerals: In commercial grape seed oil obtained via cold pressing, higher concentrations of K, Ca, Mg, P, and S were detected (832.3, 670.0, 179.4, 83.27, and 53.09 mg/100 g of sample, respectively), while lower amounts of other minerals such as Fe, Si, Al, Cl, Mn, Zn, Sr, Na, and Rb (4.47, 9.91, 13.12, 5.75, 2.75, 2.69, 1.86, 8.56, and 1.37 mg/100 g of sample, respectively) [49]. These results were comparable to those observed in other commercial seed oils obtained using the same extraction method, such as walnut (1171.5 mg of K and 665.7 mg of Ca per 100 g of sample), pomegranate (920.6 mg of K and 439.08 mg of Ca per 100 g), and almond (920.6 mg of K and 895.8 mg of Ca per 100 g) [49]. In contrast, using supercritical fluids, Parthenocissus wild grape oil has been reported to contain lower amounts of these minerals. The minerals found in the highest quantities using this extraction method were Zn, K, Ca, and Mg, with 61.4, 40.5, 40.0, and 13.3 mg/100 g of sample, respectively [84]. Although notable amounts of K, Ca, and Zn have been reported in grape seed oil obtained through pressing, compared to extraction using supercritical fluids, there is still insufficient evidence to validate these results fully.

Volatile compounds: In relation to the volatile compounds identified in grape seed oil, the analysis revealed the presence of alcohols, esters, aldehydes, terpenes, ketones, carboxylic acids, volatile phenols, lactones, and furans (see Table 5). Nonetheless, distinct volatile compounds were detected among the grape varieties depending on the pressing extraction method [57] and Soxhlet [15,61]. In one study, the primary volatile compounds identified using GC-MS in grape seed oil obtained through pressing were esters, alcohols, and aldehydes. Additionally, a greater number of these compounds was found in Cabernet Sauvignon (32 volatile compounds) compared to Chardonnay (27 volatile compounds) [57]. Another investigation found that the volatile compounds in commercial grape seed oil had pleasant aroma characteristics, such as fruity and floral notes (esters and alcohols). However, some volatile compounds contributed to unpleasant odors, including acetic acid (vinegar), isovaleric acid (sweaty, rotten smell), 2-heptanal (rancid), acetoin (buttery), and 2,4-methyl-2-hexanone (spicy, acetone) [16]. In a separate study on Okusgozu grape seed oil (red variety), which was oven-dried at 50 °C for 24 h and extracted using Soxhlet, a GC-MS-FID analysis identified 46 volatile compounds. The main families of these compounds were esters, alcohols, aldehydes, and carboxylic acids [15]. In a different study, oil extracted from the seeds of the same variety, which were dried at room temperature before Soxhlet extraction, revealed only 21 volatile compounds when analyzed using GC-MS [61]. This may be because various processes applied to grape seeds, such as thermal drying, can significantly alter their composition and the volatile compounds they contain. Regarding the volatile compounds in seed oil obtained using the Soxhlet method, it was observed that the predominant volatile compound was isoamyl acetate, an ester known for its fruity and sweet characteristics [61]. This compound was also found in the oil obtained via the pressing method [57].

These studies indicate insufficient evidence to draw definitive conclusions about the aromatic compounds in grape seed oil obtained through pressing compared to the Soxhlet method. Furthermore, it is important to highlight that the determination of volatile compounds was conducted on oils produced via conventional methods, suggesting that there is still a gap in understanding regarding the volatile compounds in grape seed oil obtained through alternative extraction methods.

#### 3.2.5. Antioxidant, Antimicrobial, Sensory, and Physicochemical Properties

Antioxidant properties: Grape seed oil compounds can slow down autoxidation or lipid degradation by inhibiting free radical formation. Common methods to assess the antioxidant activity of edible oils include 2,2-diphenyl-1-picrylhydrazyl (DDPH), 2,2′-azinobis 3-ethylbenzothiazoline-6-sulfonic acid (ABTS), oxygen radical absorbance capacity (ORAC), and ferric-reducing antioxidant power (FRAP) [96]. Several studies indicate that grape seed oil is a plentiful source of antioxidants. However, the effectiveness of this antioxidant activity varies based on the grape variety and is primarily due to the pressing extraction methods [36,43,57], including Soxhlet [33,94], ultrasound [36,53], and supercritical fluid [26,35,64]. For example, it was noted that oils extracted using supercritical fluid displayed greater antioxidant activity measured based on DPPH (with an IC_50_ of 56.69) compared to those extracted using Soxhlet (IC_50_ of 134.89) and Bligh and Dyer methods (IC_50_ of 63.68) [26]. Furthermore, optimized extraction conditions with supercritical fluids (400 bar, 41 °C, 90 min, 1.94 kg CO_2_/h) enhanced the antioxidant capacity, achieving DPPH values of up to 37.06% [35]. Conversely, a separate study revealed that oils obtained through cold pressing exhibited lower efficiency in DPPH radical elimination (ranging from approximately 43.32 to 65.18 mg of oil/mg of DPPH radical in five varieties) compared to oils extracted via ultrasound (ranging from approximately 32.96 to 42.68 mg of oil/mg of DPPH radical in five varieties) [36].

The antioxidant capacity of grape seed oil is mainly attributed to lipophilic compounds rather than hydrophilic ones, as grape seed oil contains a higher proportion of lipophilic antioxidants that are soluble in oil rather than in water [29]. Furthermore, a positive correlation has been found between lipophilic compounds (including phytosterols, vitamin E isomers like tocopherols and tocotrienols, and carotenoids) and hydrophilic compounds (such as flavonoids and phenolic acids) with their capacity to scavenge free radicals [16,52]. Regarding Barbera seed oil obtained using different extraction methods, it has been observed that oil extracted via supercritical fluids and Soxhlet methods exhibits higher lipophilic antioxidant activity than hydrophilic activity. For instance, oil extracted via supercritical fluids displayed hydrophilic antioxidant activity ranging from 0.9 to 2.0 µmol trolox/g oil and lipophilic antioxidant activity ranging from 4.9 to 8.2 µmol trolox/g oil. Similarly, the oil extracted with n-hexane from the same variety exhibited an antioxidant activity of 2.1 µmol of trolox/g and a lipophilic antioxidant activity of 6.5 µmol of trolox/g of oil [29].

On the other hand, it was observed the hydrophilic antioxidant activity of the grape seed oil from the Graševin variety is attributed to a higher extraction of phenolic compounds (hydrophilic components) through cold pressing (39.7 mg/kg) compared to supercritical fluids (23.9 mg/kg) [31]. This is attributed to a strong positive correlation between the phenolic compounds of grape seed oil and its antioxidant activity measured via DPPH [53]. On the other hand, a direct relationship has been observed between vitamin E compounds (α- and γ-tocotrienol and α-tocopherol isomers) and the antioxidant activity of grape seed oil in red varieties. Specifically, the Cornifesto (69.89%), Tinto Cão (67.83%), and Marufo (65.39%) varieties exhibited higher values of DPPH free radical scavenging, while the Trincadeira Preta variety showed the lowest value (38.68%) [88].

Antimicrobial properties: The antimicrobial properties of the oil could act as an ox-ygen entry barrier, thereby inhibiting the growth of aerobic bacteria. According to the results presented in Table 6, grape seed oil demonstrated antimicrobial efficacy against Gram-positive bacteria like *Staphylococcus aureus* [94]. However, in a recent study, it was observed that the seed oil of the Tamjanika grape variety extracted via Soxhlet had antibacterial activity against positive and negative bacteria [69]. Likewise, in another investigation, it was noted that commercial grape seed oil extracted through pressing exhibited antimicrobial efficacy against both Gram-positive bacteria (*Staphylococcus aureus*) and Gram-negative bacteria (*Escherichia coli, Salmonella typhimurium, Pseudomonas fluorescens*) [97]. Another study observed that the incorporation of grape seed oil obtained via pressing into chitosan films exhibited inhibitory effects against Gram-negative bacteria (*Stenotrophomonas maltophilia, Acinetobacter guillouiae, Pseudomonas aeruginosa, Enterobacter amnigenus*); however, the extent of inhibition varied depending on the bacterial species and strain [98]. These studies suggest that grape seed oil exhibits an inhibitory effect against Gram-positive bacteria, such as *Staphylococcus aureus*, particularly at higher concentrations. However, there is still insufficient research on Gram-negative bacteria to draw definitive conclusions.

The variation could be due to differences in the composition of the Gram-positive and Gram-negative bacterial cell wall and membrane. Antibacterial compounds found in grape seed oil, such as fatty acids (linoleic acid), tocopherols (*α*-tocopherol), phenolic compounds, and volatile compounds (carvacrol), were observed to destabilize the proteins in the phospholipid bilayers of bacterial membranes, increasing their cellular permeability [56,99,100]. This discovery had previously been noted by other researchers, who mentioned that fatty acids destabilize the phospholipid membrane, leading to bacterial inhibition [100]. This is probably because the long-chain fatty acids (linoleic acid) in grape seed oil act as anionic surfactants [94]. Furthermore, the antibacterial effects of phenolic compounds was observed, as they interact with the lipid membrane of Gram-positive bacteria, inducing structural alterations and enhancing permeability, thus disrupting bacterial homeostasis [101]. In another study, it was observed that *α*-tocopherol has also shown antimicrobial activity; however, its antioxidant capacity is more potent [102]. Recent studies observed that applying oil in the form of an emulsion reduces its particle size, improving the affinity between bacteria and the surface area, thus increasing its effectiveness as an antimicrobial agent [103]. All these findings support the idea of adding this byproduct to food products for antimicrobial purposes.

Sensory properties: Although only one report was found regarding the sensory properties of grape seed oil, it is mentioned that grape seed oil presents aromas reminiscent of pomace, wine, and raisins; however, they also perceived unpleasant attributes such as burnt oil, rubber, with a light sweet touch; in addition, they observed that aroma intensity was different between grape varieties (e.g., the Petit Verdot variety demonstrated a moderate aromatic intensity, whereas the Monastrell variety had a stronger aroma) [81]. Unpleasant characteristics in the oil are attributed to the oxidation of fatty acids and the formation of unstable hydroperoxides, which later break down into aldehydes, ketones, acids, and other volatile compounds of low molecular weight [104,105]. For instance, a study on grape seed oil identified the presence of aldehydes (2-heptenal and hexanal), carboxylic acids (acetic and isovaleric acid), and ketones (acetoin and 2,4-methyl-2-hexanone), which produce unpleasant odors and flavors, altering the sensory attributes of the oil [16].

Physicochemical properties: The peroxide value assesses lipid oxidation, a process involving free radical reactions between fatty acids and oxygen that leads to the breakdown of lipids, known as rancidity [106]. A peroxide index below 10 mEq of active oxygen/kg oil indicates higher oil quality [107]. In a recent investigation, a decrease in peroxide value was noted with increasing seed drying temperature in the Ives variety between different extraction methods: i.e., at 40 °C, pressing yielded 9.98 mEq/kg, ultrasound 28.67 mEq/kg, and Soxhlet 36.37 mEq/kg; whereas at 80 °C, pressing resulted in 6.79 mEq/kg, ultrasound 21.45 mEq/kg, and Soxhlet 18.82 mEq/kg. The decrease in the peroxide index may be linked to the oil’s rheological properties, such as viscosity and fatty acid composition. This study found that oil from seeds dried at 80 °C showed higher viscosity and slightly decreased fatty acid saturation. In comparison, oil from seeds dried at 40 °C exhibited lower viscosity and a higher level of fatty acid saturation [52]. Another study indicated lower peroxide values for cold-pressed oil (2.53–3.80 mmol/kg) in different varieties [36], while a separate investigation found ultrasound-assisted extraction produced values ranging from 13.33 to 20.47 mEq/kg across different varieties [53]. The high peroxide values obtained through ultrasound extraction might be attributed to its reliance on cavitation and mechanical action, which can lead to oil oxidation via free radical formation [108]. The high values reported by the Soxhlet method may be attributed to the long extraction time with n-hexane (6 h) and the high temperature (70 °C) (see Supplementary Materials). Hence, these studies suggest that pressing extraction (seeds dried at low temperatures) maintains superior oil quality due to its lower peroxide values.

The oil density is a physical property that measures its mass per unit volume, increasing with carbon atom count and decreasing with unsaturated bond rise. According to FAO/WHO [107], the relative density of grape seed oil should range between 0.920 and 0.926 g/mL at 20 °C. In several studies, it has been observed that the density is not affected by the extraction method or the variety and is within the range established by the FAO/WHO [107]; 0.920 g/mL [52], and 0.917–0.928 g/mL [53].

The smoking point is an indicator of the free fatty acid content within the oil, with oils containing higher levels of free or shorter-chain fatty acids displaying lower smoking points. On average, oils extracted via cold pressing exhibited a higher smoking point of 214.9 °C than those extracted using ultrasound, with a smoking point of 210.8 °C [36]. In recent research, the smoking point of grape seed oil was documented at 235 °C, being higher than corn oil (217 °C) and olive oil (192 °C), indicating its resistance to high temperatures [109].

The melting point denotes the gradual transition of fats and oils from solid to liquid. This value diminishes with the presence of unsaturated fatty acids due to their lack of a crystalline structure and interruptions in linear arrangement [110]. Oils exhibit varying melting points owing to their distinct compositions; for instance, grape seed oil, walnut oil, and safflower oil display low melting points at −48.07, −45.37, and −45.22 °C respectively, while olive oil, hazelnut oil, and peanut oil demonstrate higher melting points at −10.80, −16.87, and −20.5 °C respectively [111].

The saponification index serves as an indicator of the fatty acid chain length within the oil. A higher index implies greater potassium hydroxide (KOH) consumption, indicating the presence of shorter-chain fatty acids, whereas oils with longer-chain fatty acids exhibit lower KOH consumption, resulting in a lower saponification index. Hence, oils with higher molecular weight fatty acids display lower saponification indices. According to FAO/WHO [107], the saponification index for grape seed oil typically ranges from 188 to 194 mg KOH/g oil. Research suggests that varietal variations have a more significant influence on the saponification index compared to the extraction method. For instance, recent research revealed saponification indices of 188.83 and 193.84 mg KOH/g for Ives and Cabernet sauvignon, respectively, using the pressing method [52]. Similarly, in another study (oil extracted by pressing), varying saponification index values were observed across grape varieties: Gamay (185.0 mg KOH/g oil), Pinot noir (190.67 mg KOH/g oil), Cabernet sauvignon (187.33 mg KOH/g oil), and Merlot (191.0 mg KOH/g oil); furthermore, it was evidenced that oils obtained by pressing generally exhibited lower saponification indices compared to those extracted via ultrasound methods [36].

The iodine index is used to assess the level of unsaturation (double bonds) in oils or fatty acids. Also, it serves as an indicator of the natural oxidation process of oils. This value should fall within the 128 to 150 g I_2_/100 g [107]. This index appears to be more influenced by the grape variety than the extraction method. For instance, this is evident in red varieties such as Quebranta (120.33 g I_2_/100 g) and Mollar (122.67 g I_2_/100 g) [53], as well as Cabernet sauvignon (130.33 g I_2_/100 g), Gamay (132.0 g I_2_/100 g), Pinot noir (133.0 g I_2_/100 g), and Merlot (134.67 g I_2_/100 g) [36]. Extraction methods seem to have less impact, as similar iodine indices were found in oils extracted by cold pressing (135.50 g I_2_/100 g), Soxhlet (134.44 g I_2_/100 g), and ultrasound (134.61 g I_2_/100 g) [52]. In all three studies assessed, it was noted that grape seed oil exhibits high iodine values. This is because grape seed oil contains more than 70% polyunsaturated fatty acids, as previously reported.

The acid value indicates the quantity of free fatty acids resulting from the hydrolysis process of triglycerides. These values should be below 4 mg KOH/g oil [107], as higher values would suggest the degradation of triglyceride chains, indicating oil deterioration (hydrolytic rancidity). It has been observed that the acid values were slightly lower for varieties of grape seed oils obtained by pressing (ranging from 0.520 to 0.653 mg KOH/g) compared to those extracted using ultrasound-assisted extraction (ranging from 0.677 to 0.813 mg KOH/g) [36]. Additionally, it was noted that the grape variety can influence the acidity index of the oil. For instance, differences were found in the Syrah variety (0.82%) and Tintorera (1.42%) [41]. Similarly, seed oils obtained from five Pisco varieties exhibited acidity values ranging between 2.06 and 3.13 mg KOH/g [53]. On the other hand, it was observed that this parameter could be influenced by the temperature and drying time of the seeds. A higher acidity index was noted at lower seed drying temperatures of 40 °C (ranging from 1.83 to 2.50 mg KOH/g) compared to 80 °C (ranging from 1.23 to 2.20 mg KOH/g) [52]. This last finding would also suggest that the acidity index of grape seed oil might be changed by the methods used to obtain entire grape seeds or flour, such as drying, moisture content, and storing conditions of the seeds or flour. It is worth noting that in all the trials, the acidity value of grape seed oil was found to be less than 4 mg KOH/g, which is recommendable for a quality oil [107].

The refractive index is a parameter used to criterion the quality and purity of the oils. Likewise, it indicates the degree of hydrogenation in the oil [112]. The FAO/WHO [107], specifies that this parameter typically ranges between 1.467 and 1.477 at 40 °C. Studies indicate that the refractive index of grape seed oil remains consistent across various extraction methods and varieties. For instance, values of 1.47 were observed in all oils obtained by pressing, Soxhlet, and ultrasound in two varieties [52], while a range of 1.4751 to 1.4758 was reported for oils obtained by Soxhlet in five varieties [61], additionally, oils obtained by ultrasound in five varieties exhibited a range of 1.460 to 1.483 [53].

#### 3.2.6. Innovation of Its Application in the Food Industry

As research attention grows toward residues generated from wine production, scientific evidence confirms the dietary uses of winemaking byproducts as a functional food. Grape seed oil emerges as a focal point for researchers among these byproducts due to its diverse bioactive compounds, offering antioxidant, anti-inflammatory, antitumor, and antimicrobial properties [11,12]. Due to these attributes, this byproduct is now being utilized across diverse fields, including medical, cosmetics, food, feed, packaging, and biofuel. Therefore, this review aims to explore the compounds present in grape seed oil using different extraction methods and assess their potential applications in the food industry. Table 7 provides a summary of this information.

According to the findings presented in the previous table, this subproduct was incorporated into foods containing a substantial amount of lipids. Overall, it was observed that including grape seed oil helped inhibit lipid oxidation, extending the shelf life of the food and enhancing sensory qualities. However, excessive addition intensified the aroma and flavor of grape seed oil, resulting in reduced overall acceptability. These results highlight the necessity for further investigation into the ideal proportion of grape seed oil in such foods and its potential in novel food formulations.

## 4. Conclusions and Future Perspectives

Wine production is currently increasing, resulting in a rise in wine waste production. Consequently, researchers are concerned about the study of these residues, particularly grape seed oil, as indicated by the bibliometric analysis. Consecutively, based on the comprehensive literature review conducted, it is evident that grape seed oil is rich in bioactive compounds, providing the oil with antioxidant and antimicrobial attributes. These inherent qualities have led numerous researchers to incorporate this byproduct into novel food formulations, including yogurt, chocolate, canned fish, and sausages. Nevertheless, ensuring the quality of the oil depends on several factors, such as grape variety and the extraction process (pre-extraction, extraction, and post-extraction).

Researchers are currently investigating more environmentally friendly methods for extracting grape seed oil, aiming to achieve results comparable to or better than traditional methods in yield and oil quality. Further research is needed to optimize the conditions for each phase of the extraction process (pretreatment, extraction, and post-extraction), particularly by exploring alternative extraction methods. This includes considering the initial stages of storage and treatment of grape seeds before extraction, as these factors may affect the quality of the oil. Moreover, this review highlights the antioxidant and antimicrobial properties of grape seed oil, which have attracted significant attention from researchers for potential applications in diverse fields, including the food industry. However, it is evident that grape seed oil obtained through pressing is the only form currently utilized as an additive in food formulations. This is feasible because other traditional methods use solvents for oil extraction. These solvents can be less appropriate for food applications due to their potential toxicity and effects on the final product’s quality. Consequently, future research should use grape seed oil from alternative extraction methods, such as ultrasonic extraction, CO_2_ supercritical fluids, and pulsed electric fields. Additionally, further studies are warranted to explore the incorporation of this byproduct into novel food sources.

## Figures and Tables

**Table 1:** Fatty acid profiles (g/100 g) of different varieties of grape seed oil according to the extraction method.

Extraction Method	Palmitic Acid (C16:0)	Stearic Acid (C18:0)	Palmitoleic Acid (16:1 n-7)	Oleic Acid (C18:1 n-9)	Linoleic Acid (18:2 n-6)	*α*-Linolenic Acid (C18:3 n-3)	Variety	Ref.
	6.49	3.07	0.11	16.76	66.01	0.42	Ives	[52]
	7.99	3.78	0.11	13.01	68.75	0.40	Cabernet sauvignon	
Pressing	7.39	4.09	n.d.	13.84	74.17	0.20	Cabernet sauvignon	[36]
	7.22	3.70	n.d.	14.36	74.15	0.31	Merlot	
	6.86	3.53	n.d.	16.02	73.05	0.28	Pinot noir	
	8.88	3.33	n.d.	16.62	70.90	0.27	Sangiovese	[30]
	7.95	4.42	n.d.	22.20	64.50	0.64	Syrah	[41]
	8.47	4.60	n.d.	24.90	61.40	0.46	Tintorera	
	6.76	3.28	n.d.	16.85	72.53	0.28	Gamay *	[36]
	7.61	4.44	0.30	20.5	66.17	0.50	Graševina *	[31]
	6.83	3.22	0.14	17.68	65.25	0.48	Ives	[52]
	7.99	3.78	0.13	13.97	66.81	0.43	Cabernet sauvignon	
Soxhlet	8.97	4.04	0.09	16.75	69.00	0.44	Tempranillo	[59]
	23.50	11.04	0.10	7.23	57.68	0.16	Syrah	[26]
	7.22	3.07	0.16	16.79	72.35	0.39	Raboso Piave	[34]
	7.45	3.76	0.15	15.33	71.55	0.42	Pinot gris	[25]
	6.90	3.58	n.d.	14.13	73.11	0.39	Pinot meunier	[39]
	6.87	3.96	n.d.	14.61	72.46	0.35	Pinot noir	
	9.53	4.61	0.20	17.52	67.27	0.30	Pinot noir	[33]
	8.53	3.42	0.10	14.49	72.47	0.32	Merlot	
	10.66	4.68	0.11	14.29	69.35	0.30	Cabernet franc	
	6.87	3.87	0.19	17.14	70.15	0.45	Riesling *	[25]
	7.81	4.44	0.22	19.92	66.85	0.32	Italian Riesling *	[33]
	9.57	9.93	0.13	17.91	67.67	0.34	Rhine Riesling *	
	7.85	4.29	0.19	18.79	68.12	0.32	Sauvignon blanc *	
	6.47	4.73	n.d.	16.65	70.19	0.34	Chardonnay *	[39]
	6.80	3.14	0.15	17.39	65.25	0.48	Ives	[52]
	8.15	3.84	0.14	14.49	66.57	0.43	Cabernet sauvignon	
Ultrasound	7.59	4.29	n.d.	13.58	73.92	0.31	Cabernet sauvignon	[36]
	7.59	3.76	n.d.	13.49	74.66	0.29	Merlot	
	7.27	3.52	n.d.	15.69	73.05	0.26	Pinot noir	
	7.39	2.80	0.10	17.74	71.6	0.36	Raboso Piave	[34]
	6.26	3.66	0.07	19.70	70.07	n. d.	Moscatel *	[53]
	6.85	3.28	n.d.	16.85	72.53	0.28	Gamay *	[36]
Supercritical fluids	21.04	10.33	0.11	6.34	61.94	0.19	Syrah	[26]
	7.25	4.59	n.d.	11.91	74.82	n. d.	Cabernet franc	[35]
	6.66	4.04	n.d.	16.00	71.70	0.47	Barbera	[21]
	7.47	3.56	n.d.	15.60	71.80	0.38	Pinot noir	
	6.53	4.16	n.d.	13.60	74.30	0.43	Nebbiolo	
	6.82	3.64	n.d.	14.80	73.20	0.43	Muller Thurgau *	
	8.89	2.84	n.d.	15.30	71.0	0.56	Moscato *	
	7.62	3.55	n.d.	16.80	70.40	0.36	Chardonnay *	
	8.88	4.49	0.37	21.20	64.00	0.51	Graševina *	[31]
Pulsed electric field + supercritical fluids	8.58	4.40	0.37	21.09	64.61	0.52	Graševina *	[31]

* Refers to white grape varieties. n.d.: not detected <0.01 mg/100 g.

**Table 2:** Concentrations of tocopherol and tocotrienol (mg/kg) in seed oil from different grape varieties according to the extraction method.

	Content	Pressing	Ref.	Content	Soxhlet	Ref.	Content	Supercritical Fluid	Ref.	FAO/WHO
Tocopherols										
*α*	38.4	Merlot	[43]	24.63	Merlot	[28]	90.0	Merlot	[85]	16.0–38.0
	49.8	Merlot	[32]	39	Chardonnay *	[21]	59.1	Chardonnay *		
	47.3	Syrah	[41]	53	Chardonnay *	[29]	68	Chardonnay *	[21]	
	73	Chardonnay *	[21]	82.58	Syrah	[28]	87	Chardonnay *	[29]	
	127	Moscato *		63	Moscato *	[21]	131	Moscato *	[21]	
	41	Muller Thurgau *		27	Muller Thurgau *		51	Muller Thurgau *		
	115	Nebbiolo		114	Nebbiolo		157	Nebbiolo		
	61	Pinot noir		94	Pinot noir		79	Pinot noir		
	199	Barbera		106	Barbera		196	Barbera		
	39.8	Hamburg	[43]	75.64	Carignan	[28]	123	Moscato *	[29]	
	64.0	Ital. Riesling *		22.6	Garnacha	[82]	101	Muller Thurgau *		
	49.6	Sila *		125.1	Tempranillo		174	Nebbiolo		
	50.1	Tintorera	[41]	23.8	Tempranillo	[59]	172	Pinot noir		
	75.9	Graševina *	[31]	193.1	Palomino fino *		156	Barbera		
				188.9	Pedro Ximénez *		131.34	Cabernet sauvignon	[85]	
				172.4	Muscat Alexandria *		69.8	Graševina *	[31]	
				114.8	Tintilla de Rota					
*β*	48.4	Syrah	[41]	n.d.	Syrah	[28]	2.53	Cabernet sauvignon	[85]	n.d.–89.0
	40.5	Tintorera		10.77	Merlot		1.75	Merlot		
				41.87	Carignan		5.3	Moscato *	[29]	
				5.6	Moscato *	[29]	8.9	Muller Thurgau *		
				9.3	Muller Thurgau *		13	Nebbiolo		
				12	Nebbiolo		5.7	Pinot noir		
				6.7	Pinot noir		4.4	Barbera		
				7.4	Barbera		10.6	Chardonnay *		
				7.2	Chardonnay *		0.86	Chardonnay *	[85]	
				0.3	Tempranillo	[82]				
				0.5	Garnacha					
				1.0	Mencia					
				0.5	Carrasquín					
				0.6	Albarin *					
*γ*	12.6	Graševina *	[31]	44.7	Palomino fino *	[59]	33	Moscato *	[29]	n.d.–73.0
	24	Chardonnay *	[21]	38.6	Pedro Ximénez *		33	Muller Thurgau *		
	24	Moscato *		25.6	Muscat Alexandria *		41	Nebbiolo		
	17	Muller Thurgau *		73.3	Tintilla de Rota		43	Pinot noir		
	53	Nebbiolo		22.9	Tempranillo		31	Barbera		
	24	Pinot noir		3.4	Tempranillo	[82]	18	Chardonnay *		
	30	Barbera		3.6	Garnacha		17.37	Chardonnay *	[85]	
	16.8	Syrah	[41]	14.9	Mencia		21	Chardonnay *	[21]	
	21.5	Tintorera		14.7	Carrasquín		33	Moscato *		
				15.9	Albarin *		18	Muller Thurgau *		
				11.15	Merlot	[28]	53	Nebbiolo		
				63.84	Carignan		23	Pinot noir		
				42.77	Syrah		55	Barbera		
				20	Moscato *	[21]	40.51	Cabernet sauvignon	[85]	
				14	Muller Thurgau *		22.19	Merlot		
				51	Nebbiolo					
				25	Pinot noir					
				62	Barbera					
				11	Chardonnay *					
*δ*	1.2	Merlot	[43]	13	Moscato *	[29]	16	Moscato *	[29]	n.d.–4.0
	1.8	Hamburg		23	Muller Thurgau *		23	Muller Thurgau *		
Tocopherols										
*δ*	2.8	Ital. Riesling *	[43]	15	Nebbiolo	[29]	19	Nebbiolo	[29]	n.d.–4.0
	3.9	Sila *		32	Pinot noir		18	Pinot noir		
	n. d.	Syrah	[41]	35	Barbera		19	Barbera		
	n. d.	Tintorera		52	Chardonnay *		5	Chardonnay *		
				10.2	Palomino fino *	[59]	0.26	Chardonnay *	[85]	
				8.1	Tintilla de Rota		1.48	Cabernet sauvignon		
				11.9	Tempranillo		0.8	Merlot		
				0.5	Garnacha	[82]				
				0.9	Mencia					
				0.6	Carrasquín					
				1	Albarin *					
				0.2	Tempranillo					
Tocotrienols										
*α*	67	Moscato *	[21]	26	Moscato *	[21]	81	Moscato *	[21]	18.0–107.0
	103	Muller Thurgau *		105	Muller Thurgau *		98	Muller Thurgau *		
	167	Nebbiolo		124	Nebbiolo		170	Nebbiolo		
	75	Pinot noir		93	Pinot noir		82	Pinot noir		
	62	Barbera		68	Barbera		97	Barbera		
	131	Chardonnay *		88	Chardonnay *		122	Chardonnay *		
	215.7	Syrah	[41]	131.8	Tempranillo	[82]	177.7	Chardonnay *	[85]	
	230.8	Tintorera		85.3	Garnacha		201.59	Cabernet sauvignon		
	124.1	Graševina *	[31]	92.3	Mencia		239.95	Merlot		
				54.6	Carrasquín		124.1	Graševina *	[31]	
				73.2	Albarin *					
*β*	-	-	-	11	Moscato *	[29]	14	Moscato *	[29]	-
				20	Muller Thurgau *		18	Muller Thurgau *		
				29	Nebbiolo		32	Nebbiolo		
				8	Pinot noir		11	Pinot noir		
				12	Barbera		22	Barbera		
				14	Chardonnay *		23	Chardonnay *		
				1.3	Tempranillo	[82]	2.41	Chardonnay *	[85]	
				1.4	Garnacha		2.73	Cabernet sauvignon		
				2.1	Mencia		2.73	Merlot		
				1.1	Carrasquín					
				1.7	Albarin *					
*γ*	87	Moscato *	[21]	52	Moscato *	[21]	110	Moscato *	[21]	115.0–205.0
	198	Muller Thurgau *		187	Muller Thurgau *		212	Muller Thurgau *		
	185	Nebbiolo		154	Nebbiolo		179	Nebbiolo		
	279	Pinot noir		224	Pinot noir		253	Pinot noir		
	190	Barbera		106	Barbera		151	Barbera		
	172	Chardonnay *		131	Chardonnay *		170	Chardonnay *		
	482.5	Syrah	[41]	107	Tempranillo	[82]	128.87	Chardonnay *	[85]	
	498.3	Tintorera		80	Garnacha		164.71	Cabernet sauvignon		
	144.5	Graševina *	[31]	101.8	Mencia		156.29	Merlot		
				98	Carrasquín		99.9	Graševina *	[31]	
				101.6	Albarin *					
*δ*	16.8	Syrah	[41]	2.2	Tempranillo	[82]	11.2	Chardonnay *	[85]	n.d.–3.2
	12.6	Tintorera		1.7	Garnacha		12.07	Cabernet sauvignon		
				3.9	Mencia		0.8	Merlot		
				2.7	Carrasquín					
				3.5	Albarin *					

* Refers to white grape varieties. n.d.: not detected.

**Table 3:** Total phenol content (mg GAE/100 g oil) in seed oil from different grape varieties according to the extraction method.

Content	Pressing	Ref.	Content	Soxhlet	Ref.	Content	Ultrasound	Ref.	Content	Supercritical Fluid	Ref.
Red grape											
1.27	Merlot	[43]	97.0	Merlot	[33]	72.0	Mollar	[53]	5.51	Borgoña-Chincha	[66]
5.99	Merlot	[36]	14.85	Merlot	[58]	139.0	Quebranta		6.04	Borgoña-Ica	
15.15	Merlot	[58]	5.63	Merlot	[28]	6.99	Pinot noir	[36]	13.59	Quebranta-Chincha	
2.46	Merlot	[32]	10.6	Syrah		7.24	Gamay		12.97	Quebranta-Ica	
6.28	Cabernet sauvignon	[36]	18.26	Syrah	[58]	7.64	Prokupac		8.06	Merlot	[85]
24.0	Cabernet sauvignon	[58]	35.23	Sangiovese		6.35	Cabernet sauvignon		9.81	Cabernet sauvignon	
4.47	Hamburg	[43]	10.45	Sangiovese	[28]	5.85	Merlot		6.0	Pinot noir	[29]
6.85	Pinot noir	[36]	24.0	Pinot noir	[33]				4.7	Nebbiolo	
7.18	Gamay		5.3	Pinot noir	[29]				3.9	Barbera	
7.45	Prokupac		28.0	Cabernet franc	[33]						
14.82	Syrah	[58]	28.0	Lemberger							
17.73	Sangiovese		45.3	Cabernet sauvignon	[58]						
35.0	Carignan	[38]	11.69	Carignan	[28]						
2.93	Black Kerküş	[44]	4.2	Nebbiolo	[29]						
4.7	Verdani		3.5	Barbera							
80.0	Concord										
16.0	Ruby red										
44.0	Muscadine	[93]									
White grape											
0.93	Ital Riesling	[43]	108.0	Italian Riesling	[33]	154.0	Moscatel	[53]	4.66	Chardonnay	[85]
1.19	Sila		65.0	Rhine Riesling		59.0	Torontel		3.2	Chardonnay	[29]
10.25	Sauvignon blanc	[58]	94.3	Rhine Riesling	[94]	122.0	Albilla		2.8	Muscat	
4.34	Atfi	[44]	91.3	Welsch Riesling					4.1	Muller Thurgau	
2.82	Karfoki		61.0	Sauvignon blanc	[33]						
2.63	Kerküş		12.88	Sauvignon blanc	[58]						
2.68	Marzuna		100.5	Sauvignon blanc	[94]						
2.19	Zeyti		10.81	Muscat	[28]						
23.0	Chardonnay		2.4	Muscat	[29]						
77.0	Albariño	[90]	2.9	Chardonnay							
			73.4	Chardonnay	[94]						
			113.0	Királyleányka	[33]						
			5.81	Razagui	[28]						
			7.03	Khamri							
			6.39	Razaki							
			6.81	Marsaoui							
			3.8	Muller Thurgau	[29]						
			104.3	Smederevka	[94]						
			76.1	Tamjanika							

**Table 4:** Concentrations of phenolic compounds (ug/g oil) in grape seed oil according to the extraction method.

Compounds	Variety	Analytical Method	Pressing	Ref.	Soxhlet	Ref.	Supercritical Fluid	Ref.	PEF-SF	Ref.
Nonflavonoids										
Phenolic acid										
Gallic ac.	Graševina	HPLC-DAD/MS	0.219	[31]	-		0.033	[31]	0.583	[31]
	Manakka	HPLC-UV/Vis	-		3.70	[62]	-		-	
Hydroxybenzoic ac.	Graševina	HPLC-DAD/MS	0.473	[31]	-		0.173	[31]	0.267	[31]
*p*-OH benzoic ac.	Merlot	HPLC-DAD, ESI-QqQ-MS/MS	<0.08	[43]	-		-		-	
	Merlot	HPLC-DAD, ESI-QqQ-MS/MS	0.398	[32]	-		-		-	
	Manakka	HPLC-UV/Vis	-		2.20	[62]	-		-	
*p*-Coumaric ac.	Graševina	HPLC-DAD/MS	0.139	[31]	-		0.074	[31]	0.282	[31]
	Merlot	HPLC-DAD, ESI-QqQ-MS/MS	<0.08	[43]	-		-		-	
	Merlot	HPLC-DAD, ESI-QqQ-MS/MS	0.241	[32]	-		-		-	
	Syrah	HPLC-DAD	-		0.98	[26]	3.14	[26]	-	
Ferulic ac.	Graševina	HPLC-DAD/MS	0.084	[31]	-		0.060	[31]	0.135	[31]
	Merlot	HPLC-DAD, ESI-QqQ-MS/MS	<0.08	[43]	-		-		-	
	Merlot	HPLC-DAD, ESI-QqQ-MS/MS	0.144	[32]	-		-		-	
*trans*-Ferulic ac.	Syrah	HPLC-DAD	-		1.32	[26]	4.34	[26]	-	
Vanillic ac.	Merlot	HPLC-DAD, ESI-QqQ-MS/MS	<0.3	[43]	-		-		-	
	Merlot	HPLC-DAD, ESI-QqQ-MS/MS	0.698	[32]	-		-		-	
Proto-catechinic ac.	Merlot	HPLC-DAD, ESI-QqQ-MS/MS	<0.04	[43]	-		-		-	
	Merlot	HPLC-DAD, ESI-QqQ-MS/MS	0.073	[32]	-		-		-	
Ursolic ac.	Merlot	HPLC-DAD, ESI-QqQ-MS/MS	82.3	[43]	-		-		-	
	Merlot	HPLC-DAD, ESI-QqQ-MS/MS	136.3	[32]	-		-		-	
Chlorogenic ac.	Manakka	HPLC-UV/Vis	-		1.10	[62]	-		-	
Caffeic ac.	Syrah	HPLC-DAD	-		1.81	[26]	5.02	[26]	-	
	Manakka	HPLC-UV/Vis	-		5.20	[62]	-		-	
*trans*-Cinnamic ac.	Syrah	HPLC-DAD	-		8.48	[26]	20.65	[26]	-	
Ellagic ac.	Manakka	HPLC-UV/Vis	-		1.50	[62]	-		-	
Resveratrol	Merlot	HPLC-DAD, ESI-QqQ-MS/MS	<0.3	[43]	-		-		-	
	Merlot	HPLC-DAD, ESI-QqQ-MS/MS	2.16	[32]	-		-		-	
	Syrah	HPLC-DAD			1.04	[26]	1.25	[26]	-	
*trans*-Resveratrol	Graševina	HPLC-DAD/MS	0.084	[31]			0.069	[31]	0.111	[31]
Flavonoids										
Flavan-3-ols										
(+)-Catechin	Graševina	HPLC-DAD/MS	0.672	[31]	-		0.281	[31]	0.450	[31]
	Syrah	HPLC-DAD			12.22	[26]	3.84	[26]	-	
(-)-Epicatechin	Graševina	HPLC-DAD/MS	0.279	[31]	-		0.030	[31]	0.040	[31]
	Syrah	HPLC-DAD			18.80	[26]	2.98	[26]	-	
Procyanidin dimer B1	Graševina	HPLC-DAD/MS	1.515	[31]	-		0.773	[31]	1.338	[31]
Flavonols										
Quercetin	Graševina	HPLC-DAD/MS	0.065	[31]	-		0.063	[31]	0.107	[31]
Quercetin-3-*β*-d-glucoside	Syrah	HPLC-DAD			4.80	[26]	5.17	[26]	-	
Myricetin	Graševina	HPLC-DAD/MS	0.070	[31]	-		0.045	[31]	0.047	[31]
Kaempherol	Merlot	HPLC-DAD, ESI-QqQ-MS/MS	<0.3	[43]	-		-		-	
	Merlot	HPLC-DAD, ESI-QqQ-MS/MS	1.80	[32]	-		-		-	
Rutin	Syrah	HPLC-DAD			1.87	[26]	n. d.	[26]	-	
Flavanon (Naringenin)	Merlot	HPLC-DAD, ESI-QqQ-MS/MS	0.008	[43]	-		-		-	
	Merlot	HPLC-DAD, ESI-QqQ-MS/MS	0.125	[32]	-		-		-	
	Syrah	HPLC-DAD			5.05	[26]	13.77	[26]	-	
Flavon (Krisoeriol)	Merlot	HPLC-DAD, ESI-QqQ-MS/MS	0.023	[43]	-		-		-	
Flavonoids										
Flavon (Krisoeriol)	Merlot	HPLC-DAD, ESI-QqQ-MS/MS	0.017	[32]	-		-		-	
Biflavon (Amentoflavon)	Merlot	HPLC-DAD, ESI-QqQ-MS/MS	0.216	[43]	-		-		-	
	Merlot	HPLC-DAD, ESI-QqQ-MS/MS	0.056	[32]	-		-		-	
Isoflavon (Formononetin)	Syrah	HPLC-DAD	-		14.25	[26]	108.81	[26]	-	

Pulsed electric field (PEF), supercritical fluids (SF), high-performance liquid chromatography (HPLC), ultraviolet–visible spectroscopy (UV/Vis), diode array detector (DAD), electrospray ionization (ESI), triple quadrupole (QqQ), tandem mass spectrometry (MS/MS).

**Table 5:** Volatile compounds in grape seed oil based on extraction methods.

Method	Analytical Method	Variety	Volatile Compounds	Ref.
Pressing	GC-MS	Cabernet sauvignon	8 alcohols (ethanol, 3-methylbutanol, 2-methylbutanol, hexanol, 2,3-butandiol, 1,3-butandiol, 1-octen-3-ol, phenethyl alcohol) 8 esters (ethyl acetate, ethyl hexanoate, isoamyl acetate, 2-methyl butyl acetate, isobutyl acetate, hexyl acetate, ethyl heptanoate, ethyl octanoate) 6 aldehydes (diethylacetal, hexanal, heptanal, benzaldehyde, *trans*-2-heptenal, *trans*-2-octenal) 1 terpene (limonene) 3 ketones (3-hydroxy-2-butanone, 2-heptanone, 3-octen-2-one) 1 hydrocarbon (styrene) 3 carboxylic acids (acetic acid, hexanoic acid, octanoic acid) 1 lactone (*γ*-butyrolactone) 1 furan (2-pentylfuran)	[57]
	GC-MS	Chardonnay *	7 alcohols (ethanol, 3-methylbutanol, 2-methylbutanol, hexanol, 2,3-butandiol, 1,3-butandiol, phenethyl alcohol) 8 esters (ethyl acetate, ethyl hexanoate, isoamyl acetate, 2-methyl butyl acetate, isobutyl acetate, hexyl acetate, ethyl heptanoate, ethyl octanoate) 4 aldehydes (diethylacetal, benzaldehyde, *trans*-2-heptenal, *trans*-2-octenal) 3 ketones (3-hydroxy-2-butanone, 2-heptanone, 3-octen-2-one) 1 hydrocarbon (styrene) 3 carboxylic acids (acetic acid, hexanoic acid, octanoic acid) 1 lactone (*γ*-butyrolactone)	[57]
Soxhlet	GC-MS	Okusgozy	5 alcohols (isoamyl alcohol, 1-octen-3-ol, heptyl alcohol, phenyl-ethyl alcohol, nonanol) 4 esters (ethyl heptanoate, hexyl acetate, ethyl octanoate, ethyl laurate) 3 aldehydes (octanal, nonanal, benzaldehyde) 1 carboxylic acid (octanoic acid)	[61]
	GC-MS	Cabernet	5 alcohols (isoamyl alcohol, 1-octen-3-ol, heptyl alcohol, phenyl-ethyl alcohol, nonanol) 7 esters (isoamyl acetate, ethyl heptanoate, hexyl acetate, ethyl octanoate, benzyl acetate, phenyl ethyl acetate, ethyl laurate) 3 aldehydes (octanal, nonanal, n-decanal) 6 carboxylic acids (isovaleric acid, valeric acid, hexanoic acid, heptanoic acid, octanoic acid, nonanoic acid)	[61]
Soxhlet	GC-MS-FID	Okuzgozu	9 alcohols (3-penten-2-ol, 3-hexanol, 2-hexanol, 3-methyl cyclopentanol, 1-hexanol, 1-octen-3-ol, 2-phenyl-2-propanol, benzyl alcohol, phenyl-ethyl alcohol) 12 esters (isoamyl acetate, butyl butanoate, ethyl hexanoate, hexyl acetate, ethyl octanoate, ethyl decanoate, diethyl succinate, phenyl ethyl acetate, ethyl dodecanoate, phenoxy ethyl acetate, ethyl palmitate, ethyl linoleate) 8 aldehydes (hexanal, octanal, (*E*)-2-heptenal, nonanal, 2-nonenal, benzene acetaldehyde, (*E*,*E*)-2,4-nonadienal, (*E*,*E*)-2,4-decadienal) 1 terpene (citronellol) 3 ketones (acetoin, 2-nonanone, acetophenone) 7 carboxylic acids (pentanoic acid, hexanoic acid, heptanoic acid, octanoic acid, nonanoic acid, decanoic acid, dodecanoic acid), 2 lactones (*γ*-butyrolactone, pantolactone) 3 volatile phenols (phenol, carvacrol, 2,4-ditertbutyl phenol) 1 furan (5-phenyl-2-furanone)	[15]
	GC-MS-FID	Moscatello *	10 alcohols (3-penten-2-ol, 3-hexanol, 2-hexanol, 3-methyl cyclopentanol, 1-hexanol, 1-octen-3-ol, *α*-cumyl alcohol, butoxyethoxy ethanol, benzyl alcohol, phenyl-ethyl alcohol) 7 esters (isoamyl acetate, butyl butanoate, ethyl octanoate, ethyl benzoate, ethyl decanoate, phenyl ethyl acetate, phenoxy ethyl acetate) 3 aldehydes (hexanal, nonanal, 2-nonenal) 3 terpenes (linalool, germacrene, ∆-cadinene) 3 ketones (2-octanone, 2-nonanone, acetophenone) 5 carboxylic acids (isovaleric acid, hexanoic acid, octanoic acid, nonanoic acid, decanoic acid) 2 lactones (*γ*-butyrolactone, pantolactone) 1 volatile phenols (phenol)	[15]

* Refers to white grape varieties. Gas chromatography (GC), mass spectrometry (MS), flame ionization detector (FID).

**Table 6:** Antimicrobial properties of grape seed oil.

Extraction Method	Variety -[Oil]	Incubation Time	Gram-Positive Batteries			Gram-Negative Batteries			Ref.
Soxhlet	Tamjanika (100%)	37 °C for 24 h	*Staphylococcus aureus*	✔	DZI (8.0)	*Escherichia coli*	✘		[94]
			*Enterococcus faecalis*	✘		*Klebsiella pneumoniae*	✘		
			*Bacillus subtilis*	✘					
	Tamjanika	37 °C for 24 h	*Staphylococcus aureus*	✔	MIC (7.7), MBC (15.4)	*Pseudomonas aeruginosa*	✔	MIC (7.7), MBC (15.4)	[69]
			*Micrococcus flavus*	✔	MIC (15.4), MBC (30.8)	*Escherichia coli*	✔	MIC (7.7), MBC (15.4)	
			*Bacillus cereus*	✔	MIC (7.7), MBC (15.4)	*Enterobacter cloacae*	✔	MIC (7.7), MBC (15.4)	
Pressing	Commercial oil (50%)	37 °C for 24 h	*Staphylococcus aureus*	✘	*-*	*Escherichia coli*	✘		[49]
			*Listeria monocytogenes*	✘		*Salmonella enterica*	✘		
	Commercial oil	37 °C for 18–24 h	*Staphylococcus aureus*	✔	MIC (30), DZI (10.0)	*Escherichia coli*	✔	MIC (25), DZI (11.3)	[97]
						*Salmonella typhimurium*	✔	MIC (25), DZI (12.1)	
						*Pseudomonas fluorescens*	✔	MIC (25), DZI (11.1)	

✔: Has inhibitory action. ✘: Does not have inhibitory action; diameter of zone if inhibition mm (DZI); minimum inhibitory concentration mg/mL (MIC); minimum bacterial concentration mg/mL (MB).

**Table 7:** Grape seed oil applications in food products.

Extraction Method	Type of Food Added	Purpose of Addition to Food	Results Obtained	Ref.
Not mentioned	Added in milk for the elaboration of yogurt, in proportions of 1.5, 2.5, and 3.5%	Evaluate the antioxidant and microbiological properties of yogurt during storage for 14 days.	Increase in peroxide and acidity index in yogurt. Decrease in lactic acid batteries (*Lactobacillus bulgarus* and *Streptococcus thermophiles*) with an increase in oil.	[109]
Not mentioned	In the formulation of chocolate at four ratios of olegoel/hydrogel (0/100; 1/99; 5/95; 10/90).	Evaluate the thermal, textural, rheological, and sensory properties of chocolate.	The chocolates with the hybrid gel showed a high thermal resistance, a hardness similar to the control, low adhesiveness, and greater sensory acceptability.	[113]
Grape seed oil var. Carignan was obtained by pressing	In canned fish, only grape seed oil was added.	Evaluate the nutritional characteristics and lipid oxidation of canned sardines.	The canned fish had increased nutritional value with greater amounts of linoleic acid; the oxidation of fatty acids was also decreased.	[38]
Red grape seed oil was extracted by pressing.	For the cocoa spread formulation, 6% grape seed oil was used.	To evaluate the nutritional, physical, and sensory properties of cocoa spread.	Polyphenols and flavonoids increased in the cocoa paste, raising the antioxidant activity and intensifying grape seed oil’s aroma and flavor.	[114]
Oil was extracted by pressing.	Red mullets were submerged in a nanoemulsion with 4% grape seed oil	Evaluate the shelf-life of red mullet and the physicochemical, sensory, and microbiological characteristics.	Extended the shelf-life of red mullet fillets during cold storage because lipid oxidation and hydrolysis were slowed and microbiological contamination was reduced.	[56]
Not mentioned	In the elaboration of yogurt, in a proportion of 1.5 and 3% grape seed oil.	Evaluate physicochemical, texture, and sensory properties and produce a low-fat product.	The yogurt had a higher content of unsaturated fatty acids and hardness. However, it scored lower in relation to taste and general acceptance.	[115]
Not mentioned	Added as an emulsion in the formulation of sausages mixed with other oils (2 and 4% grape seed oil + 16% other oils).	Evaluate the physicochemical, texture, and sensory properties.	Unsaturated fatty acids increased, and saturated fatty acids decreased; hardness, elasticity, and chewiness also decreased.	[116]
Oil was extracted by pressing	Frankfurt sausages were enriched with 1, 2, 4, 6, 8, and 10% grape seed oil.	Select the best additive considering lipid oxidation and general acceptance.	The increase in grape seed oil decreases the sausages’ general acceptability. However, it decreases lipid oxidation during the 90-day storage period.	[117]

## Data Availability

No new data were created or analyzed in this study. Data sharing is not applicable to this article.

## Supplementary Materials

The following supporting information can be downloaded at: https://www.mdpi.com/article/10.3390/foods13223561/s1, Table S1: Extraction methods and yield of grape seed oil.
