# Supplementary material for: Advances in Grape Seed Oil Extraction Techniques and Their Applications in Food Products: A Comprehensive Review and Bibliometric Analysis
Source: Foods. 2024 Nov 7;13(22):3561. doi: 10.3390/foods13223561 (PMC11592970; doi:10.3390/foods13223561)
Supplement: Supplementary file 1 [file foods-13-03561-s001.zip › foods-3190763-supplementary.pdf]

foods MDPI

Table S1. Extraction methods and yield of grape seed oil (g/100 g dry weight of byproduct).

Method	Oil extraction description	%	Varieties	Ref.
Pressing	The extraction was carried out by placing 50 g of grape seed flour in a hydraulic press (Bovenau) for 72 h under 72 tons. The oil was obtained in a vial.	-	Herbemont, Seibel, Isabel Cabernet, Merlot and Muscatel	[47]
	Whole-seed oil extraction was carried out using a rotary screw press (KOMET, Expeller CA59G-CA 5963 model). The pressing capacity was 5 kg/h, the temperature of the oil obtained was 27 °C. To purify the oil, it was centrifuged at 3857 rpm for 20 min.	5.7	Syrah, Tintorera, (blend: Syrah, tempranillo, Merlot)	[41]
	Grape seed flour (<0.5 mm), var. Airen, under cold pressing with a KOMET CA59G, which had an input capacity of 5–8 kg/h.	6.1–9.1	var. Airen	[23]
	Extraction was performed using whole seeds in a KOMET screw press. The following parameters were optimized: type of grape seed pretreatment (1, 2 and 3), preheating temperature (90 and 120 °C), diameter (10 and 15 mm), screw rotation speed (40 and 70 rpm). Maximum pressing capacity was 13.9 kg/h.	-	Not Mention	[42]
	Oil extraction was carried out from grape seed flour cv. Urumchi (from China, Brazil, France), using a screw press (Model CA59G) under two treatments (seeds heated at 80 °C and without heating, for 40 min). The obtained oil was purified via centrifugation at 3500 rpm for 10 min.	14	Urumchi	[46]
	Extraction was performed using clean, whole, dry seeds via cold press with a screw press (Type, KYP20D), 5–7 kg/h capacity, 1.8 kW electric motor. At the moment the oil began to come out, it was programmed at a speed of 20 rpm, and the head of the press was heated to 40 °C. However, due to pressure and friction, the oil came out at 50 °C. The purification of the oil was carried out for three days at room temperature for natural sedimentation.	-	Merlot, Italian Riesling, Sila	[32]
	The oil was extracted using a screw press, without external heating. The purification of the oil was carried out via centrifugation at 3000 rpm for 5 min; later, it was decanted at room temperature.	4.95<br>5.65<br>5.65<br>7.70<br>7.10	Sangiovese<br>Cabernet sauvignon<br>Merlot<br>Syrah<br>Sauvignon blanc	[58]
	The extraction was carried out in a hydraulic press. The whole dry seeds were pressed at a pressure of 600 bar and a cylinder temperature of 50 °C. The oil was obtained at 30 °C.	11.6<br>11.5<br>8.8<br>10.3<br>9.6	Pinot noir<br>Gamay<br>Prokupac<br>Cabernet sauvignon<br>Merlot	[36]
	The extraction was carried out using whole seeds with a Type KYP20D screw press and a 1.8 W electric motor with a capacity of 5–7 kg/h. The screw speed was 20 rpm. Due to pressure and friction, the oil came out between 50 and 55 °C. The purification of the oil was carried out via natural sedimentation for three days at room temperature.	9.87<br>11.92<br>8.89<br>12.74	Merlot<br>Hamburg<br>Riesling<br>Sila	[43]
	The extraction was carried out using 3 kg of whole grape seeds via cold pressing Cgoldenwall CAN-G84. The obtained oil was centrifuged at 4200 rpm for 10 min at 25 °C.	7.6<br>5.5	Red grape<br>White grape	[30]
	The extraction was carried out in cycles in a hydraulic piston press, pressing 100 g of dry whole seeds under 85 tons of pressure for 300 s. For purification, a stainless-steel mesh filter was used.	13.0	Bordo	[45]
	Grape seeds were purchased from the market. The extraction was carried out prior to the pretreatment of the whole grape seed by moistening, softening by ultrasound, and enzymatic treatment. Later, extraction was carried out using a screw extrusion press (D 85-1 G).	-	Not mention	[40]
	The extraction was carried out using grape seed flour var. Carignan by cold pressing <60 °C. The purification of the oil was carried out via filtration.	7.8	Carignan	[38]
	The extraction was performed via cold pressing using an expeller press (Monforts). This was carried out per cycle using 1 kg of whole seeds. The purification of the oil was carried out by sedimentation.	6.71	Graševina	[31]
Bligh and Dyer	The extraction was carried out by weighing 10 g of sample var. Syrah with the addition of a mixed solvent chloroform, methanol, and water 10:20:8 in a conical flask. The flask was sealed and shaken for 30 min at 230 rpm on an orbital shaker. Then, 10 mL of chloroform and 1.5% sodium sulfate (10:10) were added, then stirred for two more minutes. To remove the chloroform from the oil, it was filtered through a paper filter with ammonium sulfate anhydride. Then, the chloroform was evaporated with N_2 (99.9%) in a fume hood.	10.5<br>12.7	Syrah (No pretreatment)<br>Syrah (Ultrasound 30:30 time and temperature)	[26]
Cold percolation	The extraction was carried out by placing 40 g of grape seed flour in a flask, to which 200 mL n-hexane was added. This was placed on a shaker and incubated at 25 °C for 24 h. After extraction, the residues were subjected to centrifugation at 6000 rpm at 15 °C for 10 min to separate from the liquid phase. Subsequently, the liquid phase was filtered through filter paper using a separatory funnel, and the excess n-hexane was eliminated.	14.1<br>9.6<br>14.9<br>16.6	Okuzgozu<br>Emir<br>Sangiovese<br>Moscatello	[15]

Table S1. *Cont.*

Method	Oil extraction description	%	Varieties	Ref.
Soxhlet	Soxhlet extraction was performed using 25 g sample var. Raboso piave with 300 mL of n-hexane for 6 h via continuous reflux at a maximum temperature of 70 °C. After the extraction was complete, the n-hexane was removed at 50 °C under reduced pressure with a rotary evaporator.	14.6	Raboso Piave	[34]
	The extraction was carried out using a Soxhlet extractor (SER 148/3), placing 10 g of sample in cartridges and 60 mL of n-hexane in the extraction cup. The first immersion step was boiling solvent at 69 °C for 1 h, and the washing steps were for 3 h. The solvent was recovered using a rotary evaporator at 40 °C at a low pressure of 335 mbar and rotation speed of 30 rpm. Ba: Barbera, CH: Chardonnay, MO: Moscato, NE: Nebbiolo, PI: Pinot Noir, MT: Muller Thurgau	11.0<br>14.2<br>14.7<br>12.6<br>15.5<br>11.3	Barbera<br>Chardonnay<br>Moscato<br>Nebbiolo<br>Pinot Noir<br>Muller Thurgau	[21]
	Oil was extracted using 0.5 g of grape seed flour (from 41 varieties) with n-hexane as solvent at 70 °C. Subsequently, the hexane was evaporated in a rotary evaporator at 65 °C, then dried in an oven at 60 °C until a constant weight was obtained.	3.9<br>17.3	Zenit<br>Chardonnay	[39]
	The oil extraction was carried out using a Soxhlet extractor with hexane as solvent. In addition, a heating mantle was used at 70 °C for 32 h. Subsequently, the hexane was evaporated, and the sample was weighed twice at two-day intervals until a constant weight was obtained.	17.5<br>16.3<br>13.8<br>16.9<br>11.5<br>14.9<br>15.7<br>17.1	Riesling<br>Pinot Gris<br>Pálava<br>Hibernal<br>Dornfelder<br>Blaufränkisch<br>Zweigelt<br>Laurot	[25]
	The extraction was performed using 10 g of sample var. Syrah, placing it in a cartridge and adding 150 mL of n-hexane in a round flask. The extraction was carried out in a Soxhlet extractor for 6 h and refluxed with n-hexane at the boiling temperature of the solvent (60 to 70 °C). Solvent recovery was performed in a rotary evaporator at 30 rpm and 40 °C.	12.1<br>16.1	Syrah (No pretreatment)<br>Syrah (Ultrasound 30:30 time and temperature)	[26]
	The extraction was carried out using 50 g of grape seed flour (red varieties) with 500 mL of n-hexane (1) at 74.9 °C for 6 h and petroleum ether (2) at 74.9 °C for 5 h. The solvent was removed in a vacuum rotary evaporator.	12.7 (1)<br>12.2 (2)	Red varieties	[27]
	Extraction using 10 g of grape seed flour with 170 mL of petroleum ether for 3 h at 70 °C. After the extraction was complete, the petroleum ether was evaporated.	11.08<br>10.58<br>12.67<br>10.33<br>12.02<br>10.91<br>10.47<br>9.99	Italian Riesling<br>Cabernet Franc<br>Pinot Noir<br>Sauvignon Blanc<br>Királyleányka<br>Rhine Riesling<br>Merlot<br>Lemberger	[33]
	The oil was obtained using 30 g of grape seed meal via continuous Soxhlet extraction for 8 h with 300 mL of n-hexane at 50–60 °C. The used solvent was removed using vacuum rotary evaporator at 40 °C.	8.1<br>6.8	Red grape<br>White grape	[30]
	The oil extraction was carried out using grape seed flour at 80 °C for 6 hours. Solvent removal was performed using a vacuum rotary evaporator.	15.2<br>14.3<br>11.8<br>15.8<br>12.8	Pinot noir<br>Gamay<br>Prokupac<br>Cabernet sauvignon<br>Merlot	[36]
	The extraction was carried out using Soxhlet equipment by placing 40 g of grape seed flour in cartridges with 200 mL of n-hexane. The first extraction phase was at 80 °C for 5 h, the rinsing phase was for 45 min, and the solvent recovery phase was for 30 min. Once the residual solvent was completed, it was removed using a rotary evaporator at 40 °C and at a low pressure of 2.5 kPa.	14.1<br>9.6<br>14.9<br>16.6	Okuzgozu<br>Emir<br>Sangiovese<br>Moscatello	[15]
Pressurized liquid	Extraction was performed by weighing 3 g of grape seed flour and placing it in a 33 mL cell. This was placed in the carousel of extraction (Dionex ASE 300) with a solvent extractor. Hexane was used for pressurized liquid. The extraction was programmed at 100 °C for 30 min at 1500 psi, with 60 s of purge, and 150% flushing in three cycles. Then the extract was collected in a 250 mL vial, pre-concentrated under reduced pressure, and finally dried with anhydrous sodium sulfate.	-	Herbemont, Seibel, Isabel Cabernet, Merlot and Muscatel	[47]
Ultrasonic (with solvent)	A total of 25 g of sample var. Raboso piave with 200 mL of n-hexane was placed in a beaker. Ultrasound was applied to the beaker submerged in an ice bath for 30 min at a frequency of 20 KHz and 50 (1), 100 (2), and 150 (3) W for 30 min (maintaining temperature below 30 °C). After extraction, it was filtered, and the solvent was removed in a rotary evaporator at 50 °C.	11.42<br>13.13<br>14.08	Raboso Piave	[34]

Table S1. *Cont.*

Method	Oil extraction description	%	Varieties	Ref.
Ultrasonic (with solvent)	A total of 50 g of grape seed flour and 100 mL of n-hexane were mixed in a flask, then ultrasound was applied for 90 min at a frequency of 35 KHz and a power of 150 W, controlling the temperature at 30 °C. Then, the solid phase was separated via decantation and washed with n-hexane.	12.8<br>13.0<br>10.1<br>11.7<br>11.5	Pinot noir<br>Gamay<br>Prokupac<br>Cabernet sauvignon<br>Merlot	[36]
	Ground seeds (5 g) from Burgundy grapes were extracted in a 0.25 L Erlenmeyer flask using Ultrasound Cleaner 800 (40 KHz, 25 °C). Various extraction durations (30, 60, 90 min) and seed/solvent ratios (1:4, 1:6, 1:8) were utilized with hexane (HE), ethyl acetate (EA), and dichloromethane (DCM). Finally, the solvents were removed. The treatments were HE: 90 min, 1:8 (1), EA: 90 min, 1/8 (2), and DCM: 90 min, 1/8 (3)	11.6 (1)<br>12.6 (2)<br>12.9 (3)	Not mention	[37]
	The extraction was carried out in an ultrasound bath (UltraCleaner 800) at 40 KHz, at 25 °C for 1.5 h, using 5 g of grape seed flour and 40 mL of dichloromethane as a solvent. The seeds were previously dried at 40 °C (1) and 80 °C (2) before grinding.	12.6–11.2<br>11.2–11.9	Cabernet sauvignon<br>Ives	[52]
Supercritical fluids (CO_2)	The extraction was carried out in a 0.1 L basket. For each test, 65 g of grape seed flour was used. The extractions were carried out at a pressure of 500 bar and at 50 °C; the solvent flow rate was set at 8 g/min.	10.9<br>13.8<br>12.6<br>10.9<br>15.5<br>10.1	Barbera<br>Chardonnay<br>Moscato<br>Nebbiolo<br>Pinot Noir<br>Muller Thurgau	[21]
	The extraction was carried out by placing 65 g of grape seed flour in a 0.1 L capacity extractor. The extraction was carried out at 50 °C, a pressure of 500 bar, and a CO_2 flow rate of 8 g/min.	-	Barbera<br>Chardonnay<br>Moscato<br>Nebbiolo<br>Pinot Noir<br>Muller Thurgau	[29]
	The extraction was carried out by placing 100 g of sample into an extractor container. The optimized conditions for the extraction were as follows: temperature 41 °C, pressure 40 MPa, CO_2 flow 1.94 kg/h, extraction time 1.5 h.	14.49	Cabernet Franc	[35]
	The extraction consisted of placing 60 g of sample in each extraction cell. The conditions used for the extraction were as follows: constant temperature 50 °C, pressure 50 MPa, CO_2 flow 6 g/min. The total extraction time was approximately 1.5 h.	12.2<br>13.9	Syrah (No pretreatment)<br>Syrah (Ultrasound 30:30 time and temperature)	[26]
	Supercritical fluids (CO_2)<br>A total of 30 g of sample was used. The oil was extracted under the following conditions: pressure 25 MPa and 40 °C, for 3 h.	10.3	Red grape	[27]
	Supercritical fluids (CO_2-expanded ethanol)<br>For the extraction, 30 g of sample was used and ethanol-expanded with CO_2, which was pumped for supercritical extraction at a pressure of 7.4 MPa, a temperature of 40 °C, and a molar fraction of CO_2 of 0.3 for 2 h.	13.6	Red grape	[27]
	The extraction was carried out by measuring 35 g of grape seed flour var. Quebranta. The grapes came from three growing areas: A (San Juan Bautista), B (Subtanjalla), and C (Los Patos), the city of Pisco. This flour was placed inside the extraction cells. The extraction conditions were at 35.5 °C and 188 bar of pressure with a CO_2 flow of 47 g/min.	16.7 (A)<br>17.0 (B)<br>16.17 (C)	Quebranta	[64]
	The extraction was carried out using 30 g of sample. The extraction conditions were 30 MPa and 50 °C for 90 min at a flow rate of 1.5 kg/h.	18.5	*Parthenocissus* wild grape	[84]
	Prior to drying (35 °C, 48 h) and grinding the grape seeds, 50 g of flour was placed in a 100 mL stainless steel cylindrical extractor. CO_2 extraction was performed for 90 min. The other parameters were optimized as follows: pressure (30, 40 and 50 MPa), temperature (35, 45 and 55 °C), and flow rate (15, 30 and 45 g/min).	6.73–7.86	Graševina	[31]
Pulsed electric fields + supercritical fluids	Prior to the extraction with supercritical fluids, a pretreatment with a pulsed electric fields was carried out, for which 70 g of flour was used. After, the pretreatment it was lyophilized. Then, 50 g of the lyophilized sample was used to perform the extraction with CO_2 supercritical fluids.	7.84–8.17	Graševina	[31]

References

15. Sevindik, O.; Kelebek, H.; Rombolà, A.D.; Selli, S. Grape Seed Oil Volatiles and Odour Activity Values: A Comparison with Turkish and Italian Cultivars and Extraction Methods. *J. Food Sci. Technol.* **2022**, *59*, 1968–1981. https://doi.org/10.1007/s13197-021-05212-3.
21. Fiori, L.; Lavelli, V.; Duba, K.S.; Sri Harsha, P.S.C.; Mohamed, H.B.; Guella, G. Supercritical CO_2 Extraction of Oil from Seeds of Six Grape Cultivars: Modeling of Mass Transfer Kinetics and Evaluation of Lipid Profiles and Tocol Contents. *J. Supercrit. Fluids* **2014**, *94*, 71–80. https://doi.org/10.1016/j.supflu.2014.06.021.
23. Fernández, C.M.; Ramos, M.J.; Pérez, Á.; Rodríguez, J.F. Production of Biodiesel from Winery Waste: Extraction, Refining and Transesterification of Grape Seed Oil. *Bioresour. Technol.* **2010**, *101*, 7019–7024. https://doi.org/10.1016/J.BIORTECH.2010.04.014.
25. Burg, P.; Masán, V.; Rutkowski, K.; Burgová, J.; Lampíř, L.; Višacki, V. Seed Oil Content and Selected Qualitative Parameters of Oils from Grape Seeds. *Potravin. Slovak. J. Food Sci.* **2017**, *11*, 629–633. https://doi.org/10.5219/810.
26. De Souza, R.d.C.; Souza Machado, B.A.; Barreto, G.d.A.; Lessa Leal, I.; Jeancarlo, P. dos A.; Umsza-Guez, M.A. Effect of Experimental Parameters on the Extraction of Grape Seed Oil Obtained by Low Pressure and Supercritical Fluid Extraction. *Molecules* **2020**, *25*, 1634. https://doi.org/10.3390/molecules25071634.
27. Li, H.; Fu, X.; Deng, G.; David, A.; Huang, L. Extraction of Oil from Grape Seeds (*Vitis vinifera* L.) Using Recyclable CO_2-Expanded Ethanol. *Chem. Eng. Process.—Process Intensif.* **2020**, *157*, 108147. https://doi.org/10.1016/j.cep.2020.108147.
29. Mohamed, H.B.; Duba, K.S.; Fiori, L.; Abdelgawed, H.; Tlili, I.; Tounekti, T.; Zrig, A. Bioactive Compounds and Antioxidant Activities of Different Grape (*Vitis vinifera* L.) Seed Oils Extracted by Supercritical CO_2 and Organic Solvent. *LWT* **2016**, *74*, 557–562. https://doi.org/10.1016/J.LWT.2016.08.023.
30. Di Stefano, V.; Bongiorno, D.; Buzzanca, C.; Indelicato, S.; Santini, A.; Lucarini, M.; Fabbrizio, A.; Mauro, M.; Vazzana, M.; Arizza, V.; et al. Fatty Acids and Triacylglycerols Profiles from Sicilian (Cold Pressed vs. Soxhlet) Grape Seed Oils. *Sustainability* **2021**, *13*, 13038. https://doi.org/10.3390/su132313038.
31. Ćurko, N.; Lukić, K.; Tušek, A.J.; Balbino, S.; Vukušić Pavičić, T.; Tomašević, M.; Redovniković, I.R.; Ganić, K.K. Effect of Cold Pressing and Supercritical CO_2 Extraction Assisted with Pulsed Electric Fields Pretreatment on Grape Seed Oil Yield, Composition and Antioxidant Characteristics. *LWT—Food Sci. Technol.* **2023**, *184*, 114974. https://doi.org/10.1016/j.lwt.2023.114974.
32. Bjelica, M.; Vujasinović, V.; Rabrenović, B.; Dimić, S. Some Chemical Characteristics and Oxidative Stability of Cold Pressed Grape Seed Oils Obtained from Different Winery Waste. *Eur. J. Lipid Sci. Technol.* **2019**, *121*, 1800416. https://doi.org/10.1002/EJLT.201800416.
33. Kapcsándi, V.; Hanczné Lakatos, E.; Sik, B.; Linka, L.Á.; Székelyhidi, R. Characterization of Fatty Acid, Antioxidant, and Polyphenol Content of Grape Seed Oil from Different *Vitis vinifera* L. Varieties. *OCL* **2021**, *28*, 30. https://doi.org/10.1051/OCL/2021017.
34. Da Porto, C.; Porretto, E.; Decorti, D. Comparison of Ultrasound-Assisted Extraction with Conventional Extraction Methods of Oil and Polyphenols from Grape (*Vitis vinifera* L.) Seeds. *Ultrason. Sonochem* **2013**, *20*, 1076–1080. https://doi.org/10.1016/J.ULTSONCH.2012.12.002.
35. Jokić, S.; Bijuk, M.; Aladić, K.; Bilić, M.; Molnar, M. Optimisation of Supercritical CO_2 Extraction of Grape Seed Oil Using Response Surface Methodology. *Int. J. Food Sci. Technol.* **2016**, *51*, 403–410. https://doi.org/10.1111/ijfs.12986.
36. Milanović, J.; Malićanin, M.; Rakić, V.; Jevremović, N.; Karabegović, I.; Danilović, B. Valorization of Winery Waste: Prokupac Grape Seed as a Source of Nutritionally Valuable Oil. *Agronomy* **2021**, *11*, 1864. https://doi.org/10.3390/AGRONOMY11091864.
37. de Menezes, M.L.; Johann, G.; Diório, A.; Curvelo Pereira, N.; da Silva, E.A. Phenomenological Determination of Mass Transfer Parameters of Oil Extraction from Grape Biomass Waste. *J. Clean. Prod.* **2018**, *176*, 130–139. https://doi.org/10.1016/j.jclepro.2017.12.128.
38. Bouriga, N.; Rjiba Bahri, W.; Mili, S.; Massoudi, S.; Quignard, J.P.; Trabelsi, M. Variations in Nutritional Quality and Fatty Acids Composition of Sardine (Sardina Pilchardus) during Canning Process in Grape Seed and Olive Oils. *J. Food Sci. Technol.* **2022**, *59*, 4844–4852. https://doi.org/10.1007/S13197-022-05572-4/TABLES/5.
39. Lachman, J.; Hejtmánková, A.; Táborský, J.; Kotíková, Z.; Pivec, V.; Střalková, R.; Vollmannová, A.; Bojňanská, T.; Dědina, M. Evaluation of Oil Content and Fatty Acid Composition in the Seed of Grapevine Varieties. *LWT—Food Sci. Technol.* **2015**, *63*, 620–625. https://doi.org/10.1016/J.LWT.2015.03.044.
40. Sun, L.; Wang, H.; Wei, J.; Xue, Y.; Lan, S.; Li, X.; Yu, D.; Wang, J. Extracting Oil from Grape Seed Using a Combined Wet Enzymatic Process and Pressing. *Innov. Food Sci. Emerg. Technol.* **2022**, *77*, 102941. https://doi.org/10.1016/J.IFSET.2022.102941.
41. Navas, P.B. Composición Química Del Aceite Virgen Obtenido Por Extracción Mecánica de Algunas Variedades de Uva (*Vitis vinifera* L.) Con Énfasis En Los Componentes Minoritarios. *Arch. Latinoam. Nutr.* **2009**, *59*, 214–219.
42. Rombaut, N.; Savoire, R.; Thomasset, B.; Castello, J.; Van Hecke, E.; Lanoisellé, J.L. Optimization of Oil Yield and Oil Total Phenolic Content during Grape Seed Cold Screw Pressing. *Ind. Crops Prod.* **2015**, *63*, 26–33. https://doi.org/10.1016/J.INDCROP.2014.10.001.
43. Vujasinović, V.B.; Bjelica, M.M.; Čorbo, S.C.; Dimić, S.B.; Rabrenović, B.B. Characterization of the Chemical and Nutritive Quality of Coldpressed Grape Seed Oils Produced in the Republic of Serbia from Different Red and White Grape Varieties. *Grasas Y Aceites* **2021**, *72*, e411. https://doi.org/10.3989/gya.0222201.
45. Dalposso, P.V.; de Aguiar, C.M.; Torquato, A.S.; Tiuman, T.S.; Martin, C.A.; Zara, R.F.; Cottica, S.M. Optimization of Antioxidant Extraction and Characterization of Oil Obtained by Pressing Cold from *Vitis labrusca* Seeds. *Food Sci. Technol.* **2022**, *42*, e47420. https://doi.org/10.1590/FST.47420.

46. Zhao, B.; Gong, H.; Li, H.; Zhang, Y.; Lan, T.; Chen, Z. Characterization of Chinese Grape Seed Oil by Physicochemical Properties, Fatty Acid Composition, Triacylglycrol Profiles, and Sterols and Squalene Composition. *Int. J. Food Eng.* **2019**, *15*, 20190031. https://doi.org/10.1515/ijfe-2019-0031.
47. dos Santos Freitas, L.; Jacques, R.A.; Richter, M.F.; da Silva, A.L.; Caramão, E.B. Pressurized Liquid Extraction of Vitamin E from Brazilian Grape Seed Oil. *J. Chromatogr. A* **2008**, *1200*, 80–83. https://doi.org/10.1016/j.chroma.2008.02.067.
52. de Menezes, M.L.; Johann, G.; Diório, A.; Schuelter Boeing, J.; Visentainer, J.V.; Raimundini Aranha, A.C.; Curvelo Pereira, N. Comparison of the Chemical Composition of Grape Seed Oil Extracted by Different Methods and Conditions. *J. Chem. Technol. Biotechnol.* **2023**, *98*, 1103–1113. https://doi.org/10.1002/JCTB.7314.
58. Konuskan, D.B.; Kamiloglu, O.; Demirkeser, O. Fatty Acid Composition, Total Phenolic Content and Antioxidant Activity of Grape Seed Oils Obtained by Cold- Pressed and Solvent Extraction. *Indian. J. Pharm. Educ. Res.* **2019**, *53*, 144–150. https://doi.org/10.5530/ijper.53.1.19.
64. Barriga-Sánchez, M.; Rosales-Hartshorn, M. Effects of Subcritical Water Extraction and Cultivar Geographical Location on the Phenolic Compounds and Antioxidant Capacity of Quebranta (*Vitis vinifera*) Grape Seeds from the Peruvian Pisco Industry by-Product. *Food Sci. Technol.* **2022**, *42*, e107321. https://doi.org/10.1590/FST.107321.
84. Shakhbanov, K.S.; Abdulagatov, I.M.; Aliev, A.M. Supercritical Carbon Dioxide Extraction of Parthenocissus Wild Grape Seed Fatty Oil. *Russ. J. Phys. Chem. B* **2022**, *16*, 1213–1217. https://doi.org/10.1134/S1990793122070144/TABLES/4.
